# Traditional and new trend strategies to enhance pigment contents in microalgae

**DOI:** 10.1007/s11274-024-04070-3

**Published:** 2024-07-20

**Authors:** Aitor Aizpuru, Armando González-Sánchez

**Affiliations:** 1 Universidad del Mar, Campus Puerto Ángel, San Pedro Pochutla, 70902 Oaxaca, Mexico; 2 Instituto de Ingeniería, Universidad Nacional Autónoma de México, Ciudad Universitaria, Circuito Escolar, 04510 Mexico City, Mexico

**Keywords:** Microalgae, Chlorophylls, Carotenoids, Phycobiliproteins, Polyphenols, Flavonoids

## Abstract

Microalgae are a source of a wide variety of commodities, including particularly valuable pigments. The typical pigments present in microalgae are the chlorophylls, carotenoids, and phycobiliproteins. However, other types of pigments, of the family of water-soluble polyphenols, usually encountered in terrestrial plants, have been recently reported in microalgae. Among such microalgal polyphenols, many flavonoids have a yellowish hue, and are used as natural textile dyes. Besides being used as natural colorants, for example in the food or cosmetic industry, microalgal pigments also possess many bioactive properties, making them functional as nutraceutical or pharmaceutical agents. Each type of pigment, with its own chemical structure, fulfills particular biological functions. Considering both eukaryotes and prokaryotes, some species within the four most promising microalgae groups (Cyanobacteria, Rhodophyta, Chlorophyta and Heterokontophyta) are distinguished by their high contents of specific added-value pigments. To further enhance microalgae pigment contents during autotrophic cultivation, a review is made of the main related strategies adopted during the last decade, including light adjustments (quantity and quality, and the duration of the photoperiod cycle), and regard to mineral medium characteristics (salinity, nutrients concentrations, presence of inductive chemicals). In contrast to what is usually observed for growth-related pigments, accumulation of non-photosynthetic pigments (polyphenols and secondary carotenoids) requires particularly stressful conditions. Finally, pigment enrichment is also made possible with two new cutting-edge technologies, via the application of metallic nanoparticles or magnetic fields.

## Introduction

Microalgae are photosynthetic unicellular organisms, found in freshwater or marine environments. The term “microalgae” encompasses both eukaryotic microalgae and more primitive prokaryotic Cyanobacteria (Saini et al 2020). Despite the manifest biological differences between these microorganisms, such as the lack of membrane-bound structures in Cyanobacteria, the physiology, biotechnological applications and generated added-value products are analogous between both cells (Garrido-Cardenas et al 2018). Among the wide number of microalgal species, only a few have been thoroughly studied and are used in the industry, leaving a huge potential for the discovery of new strains, applications, and derivative compounds (Garrido-Cardenas et al 2018).

The whole biomass was the first product obtained from microalgae (Garrido-Cardenas et al 2018). It is still one of the most important outputs in terms of the amount produced and its economic value, having applications in human nutrition, and, to a lesser extent, in husbandry and aquaculture feed, or as soil fertilizer (Levasseur et al 2020). However, microalgal biomass can be treated via a biorefinery process to obtain specific industrial derivative products such as lipids, carbohydrates, proteins, and pigments (Patel et al 2022). Particularly, pigments, as the most added-value derivatives, play a decisive role in microalgal biomass value, with applications as natural food colorants, health food additives, cosmetic and pharmaceutical components, and molecular reagents (Velasco et al 2023; Tounsi et al 2023; Razzak 2024). Algae-derived pigments generally exhibit higher antioxidant bioactivity than plant-based pigments, and are healthier than synthetic ones (Cao et al 2023).

Microalgae pigments are organic molecules containing a chromophore composed of long chains or closed rings of conjugated carbon double bonds that can characteristically absorb specific regions of the visible spectrum (Saini et al 2020; Patel et al 2022). They are classified into four groups: chlorophylls, carotenoids, phycobiliproteins and more recently polyphenols. Among these pigments, some play a photosynthetic role, by directly transforming light energy into chemical energy and microalgal biomass (primary photosynthetic pigments), or by expanding the range of harvested solar wavelengths (auxiliary photosynthetic pigments). Other pigments are non-photosynthetic, and usually play a protective antioxidant role.

Photosynthetic pigments are present in the thylakoid structures, located in the chloroplast or in the cytoplasm of eukaryotic and prokaryotic microalgae, respectively. The two photosystems present in the thylakoids (photosystem I and II) have a reaction center and a light-harvesting antenna complex. Among the photosynthetic pigments, one particular type of chlorophyll, chlorophyll *a* (Chl *a*), plays a preponderant role in the reactions centers, as it is responsible for the ultimate transfer of light energy to the photosynthetic electron transport chain (PETC), where it is converted into chemical energy (Mulders et al 2014). As such, Chl *a* is referred to as the primary photosynthetic pigment. However, it has recently been found that, in very particular cases, the primary photosynthetic pigment can be another chlorophyll, Chl *d*, as it is for the Cyanobacteria *Acaryochloris marinus* (Simkin et al 2022). The antenna complex, surrounding the reactions centers, contains auxiliary photosynthetic pigments which absorb light and channel the excitation energy to the primary photosynthetic pigment, increasing the absorption cross section of reaction centers. Chl *a*, as well as other chlorophylls and other pigments such as carotenoids and phycobiliproteins, called as auxiliary photosynthetic pigments, are constituents of the light-harvesting antenna complexes expanding the range of light wavelengths absorbed and used for photosynthesis (da Silva and Lombardi 2020).

Non-photosynthetic pigments are also present in microalgae, they are classified as secondary pigments. They generally have a photoprotective function, and are located outside the thylakoids. Polyphenols are typical secondary pigments, as well as some carotenoids which are present outside of the photosynthetic apparatus.

In the first part of this review, each of the four classes of pigments encountered in microalgae is comprehensively and separately described. Particular focus is given to the commercial and potential applications of each group of pigments, as well as to their principal functions within the microalgal cell. Their characteristic chemical structure, and the main microalgal sources known to date are also presented. Finally, as the composition of microalgae, and particularly its pigments contents, can be highly influenced by cultivation conditions, strategies for pigment enhancement are reviewed and discussed. Enhancement of pigment content is typically obtained by adjusting the light characteristics (intensity and wavelength) and the mineral medium composition (Saini et al 2020; Ashokkumar et al 2023). Particularly determining conditions include cultivation under nutrient limitations, or under the presence of a high medium salinity, of chemical inductive agents, or of metal salts (Ambati et al 2018; Liang et al 2019; Levasseur et al 2020). Two explorative pigment enhancement strategies are finally discussed, the application of magnetic fields during microalgal cultivation, or the presence of nanoparticles (Vargas-Estrada et al 2020; Santos et al 2022).

## Different types of microalgal pigments

### Chlorophylls

Chlorophylls are necessary for photosynthesis, and are present in all photoautotrophic organisms (Saini et al 2020). They are commonly used in the food industry (as green dyeing agents, additive E140(i)), and in pharmaceutical and cosmetic preparations for their antioxidant, antimutagenic and antimicrobial properties, among other health benefits (Levasseur et al 2020). Although green microalgae can be a source of more chlorophyll content than terrestrial plants (e.g. 6.7% of the dry weight (DW) of *Chlorella vulgaris* (Chlorophyta) (Nakanishi and Deuchi 2014)), plants (mainly grass or alfalfa) remain the major natural sources of chlorophyll, as their production is the most cost-effective (Deepika et al 2022). Thus, the production of microalgae chlorophylls is of limited commercial interest, and should only be considered as part of a multiple compound’s valorization during biorefinery processes (Nakanishi and Deuchi 2014; Levasseur et al 2020; Chen et al 2022).

**Fig. 1:**
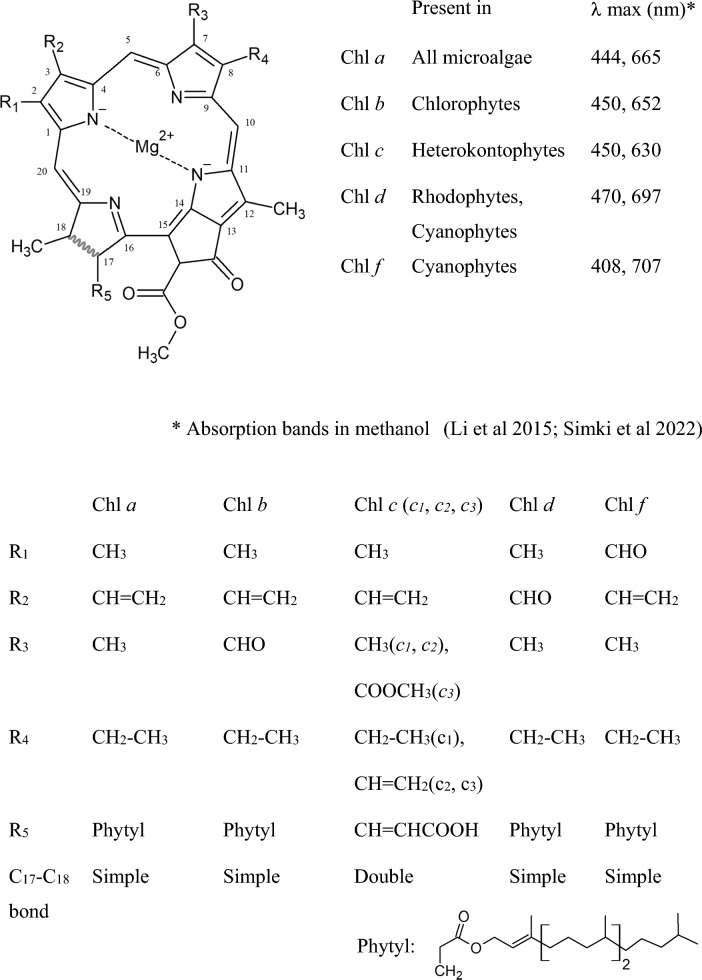
The structure and the maximum absorption bands of chlorophylls

Chlorophylls consists of four pyrroles (4 carbons and 1 nitrogen ring, C_4_H_5_N) connected by one-carbon bridges to form a conjugated tetrapyrrole ring, with a fused modified cyclopentanone. The center of this tetrapyrrole ring holds a magnesium ion (Cao et al 2023). The chemical structure of the five existing forms of chlorophyll (Chl *a*,* b*,* c*,* d*, and *f*) differs according to their C_17_-C_18_ carbon bond, and to five side chain substituents (R_1_, R_2_, R_3_, R_4_, and R_5_) in the tetrapyrrole ring, respectively at the C_2_, C_3_, C_7_, C_8_ and C_17_ carbon positions (Fig. 1).

Chl *a*, the predominant form of chlorophyll, is present in the photosynthetic reaction center of photosynthetic organisms, and in the antenna complex. Chl *b* is also present, as auxiliary pigment, in the antenna complexes of all green microalgae (Chlorophyta), but absent in red microalgae (Rhodophyta), which instead contain Chl *d* (da Silva and Lombardi 2020). Other microalgae (Heterokontophyta), particularly including brown microalgae (Phaeophyceae) and diatoms (Bacillariophyceae) use Chl *c* as an auxiliary photosynthetic harvesting pigment. It is worth noting that Chl *d* has been reported to be present in a Cyanobacteria (*Acaryochloris marina*) accounting for more than 95% of its total Chl content. Chl *f* was discovered in 2010 in the Cyanobacteria *Halomicronema hongdechloris* (Cao et al 2023).

All chlorophylls absorb the light in two main regions of the visible spectrum, exhibiting bands of absorption in the blue (≈400–470 nm) and the red regions (≈630–700 nm), respectively called the Soret and Q bands. The lack of absorption in the green region (between blue and red) explains their greenish color (Li and Chen 2015). The structural variances between the different forms of chlorophylls shift their respective absorption spectra (Deepika et al 2022) (Fig. 1). The maximum chlorophyll absorption in the Q band (dissolved in methanol) is around 665 (Chl *a*), 652 (Chl *b*), 630 (Chl *c*), 697 (Chl *d*), and 707 nm (Chl *f*) (da Silva and Lombardi 2020). Chl *f* is the one form of chlorophyll that absorbs at the higher (Q band) and the lower (Soret band) wavelengths. This wider absorption spectrum of Chl *f* can increase its photon utilization and thusly increase the biomass (Cao et al 2023).

### Carotenoids

Carotenoids are produced by all photosynthetic organisms, including all microalgae, and by some non-photosynthetic organisms (several types of bacteria and fungi) (Ashokkumar et al 2023; Simkin et al 2022). They are the most diverse class of pigments, with more than 600 natural compounds identified, including 50 varieties in algae (Levasseur et al 2020; Saini et al 2020). Carotenoids are grouped into two families, the carotenes which contain carbon and hydrogen, and the xanthophylls, which also contain oxygen. Only a few carotenoids are actually commercialized, including two carotenes (β-carotene and lycopene) and four xanthophylls (astaxanthin, canthaxanthin, lutein and zeaxanthin), all of which are present in microalgae (Ashokkumar et al 2023; Gong and Bassi 2016; Razzak 2024). Together, β-carotene and astaxanthin represent nearly 50% of the current carotenoid market (Gong and Bassi 2016).

Carotenoids, the most commercialized class of biological pigments, have many applications. First of all, β-carotene, a vitamin A precursor, is essential in our diet, to support vision, skin health, and immunity (Ashokkumar et al 2023). Furthermore, some pharmaceutical formulations include carotenoids to provide protection from the harmful effects of oxidative stress (Razzak 2024). Other related health benefits include the prevention of compromised immune response, of premature aging, and of certain cancers (Saini et al 2020). More specifically, lycopene, lutein and canthaxanthin are recognized for their respective regulation of the formation of atheromatous, cardiovascular and blood-related disorders (Saini et al 2020). In the nutraceutical eye health market, lutein and zeaxanthin are gaining importance, as they can prevent cataract and macular degeneration (Gong and Bassi 2016). Carotenoids are also widely used as colorant additives for human food, with the E160 international numbering being applicable to carotenes (carotene: E160a; lycopene: E160d), and the E161 numbering being applicable to xanthophylls (lutein: E161b; zeaxanthin: E161h; canthaxanthin: E161g; astaxanthin: E161j) (Coulombier et al 2021). Additionally, lutein, zeaxanthin, canthaxanthin and astaxanthin have been added to animal feed formulations (for poultry and farm-raised salmonids), to enhance the color of the egg-yolks and muscle tissue (Gupta et al 2021).

As an alternative to synthetic pigments, natural carotenoids are also used in cosmetic products. Particularly, some lipsticks contain lycopene and astaxanthin to provide bright red and vibrant red–orange colors (Razzak 2024). The antioxidant properties of lycopene have been exploited by the cosmetics industry, particularly for so-called anti-ageing formulations (Levasseur et al 2020). Similarly, cosmetic foundations containing β-carotene or lutein, with alleged skin rejuvenating properties, provide yellow-to-orange and light yellow-golden tones, respectively. Moreover, astaxanthin (the most potent antioxidant present in nature (Saini et al 2020)), has photoprotective properties that have been exploited via its use as sunscreen and UV protection ingredients (Razzak 2024).

Microalgal carotenoids can be classified as primary or secondary via reference to whether they have a photosynthetic or a non-photosynthetic role. Notwithstanding, lycopene plays a peculiar role, as it is the precursor of all primary and secondary carotenoids (Mulders et al 2015). Primary carotenoids (e.g. α and β-carotene, lutein and zeaxanthin), which are greater in content under normal cell growth conditions (Shi et al 2020), are necessary for adequate photosynthesis and cell survival, as structural and functional components of the photosynthetic apparatus (Eonseon et al 2003; Pagels et al 2020a). Actually, carotenoid/chlorophyll binding protein complexes are present in thylakoid membranes of Cyanobacteria and eukaryotic algae. Particularly, β-carotene is found in the core complexes of photosystem I and II (Chen et al 2020; Deepika et al 2022), while lutein and zeaxanthin are bound to the antenna proteins of the light harvesting complexes (Zheng et al 2022; Simkin et al 2022). Besides regulating and stabilizing the photosystems structures of microalgae, primary carotenoids have key photosynthetic roles (Eonseon et al 2003; Deepika et al 2022). First of all, carotenoids are indispensable for harvesting light and transferring its energy during photosynthesis (Chen et al 2020). With a light absorption range between 400 and 550 nm, including the green window not absorbed by chlorophylls (Deepika et al 2022) (Table 1), carotenoids act as accessory photosynthetic pigments. They broaden the spectrum of collected light, absorbing photons and finally transferring the excitation energy to the primary Chl *a* (Simkin et al 2022; Pagels et al 2020a). As such it is estimated that carotenoids are responsible for ~20–30% of the light harvested during this process (Deepika et al 2022). Additionally, primary carotenoids can also protect the photosynthesis apparatus from photooxidative damage (Chen et al 2020; Cano et al 2021). When chlorophylls receive more light than they can transfer to the PETC, they can elevate their energy from a basal singlet spin state to a triplet excited state. In turn, such triplet Chl can transfer its energy to ground state molecular oxygen, forming reactive oxygen species (ROS), among which is highly reactive singlet oxygen (Mulders et al 2014). ROS cause cell damage in the vicinity of their production area, generating protein oxidations, and chloroplasts and thylakoids membranes disruptions (Coulombier et al 2021). Thus, in excessive light, damage to the photosystems occurs, reducing photosynthetic capacity, a process known as photoinhibition (Simkin et al 2022). Some carotenoids can directly neutralize singlet oxygen when formed (Coulombier et al 2021). For instance, the *β*-carotene present in the core complex of photosystems quenches triplet Chl and singlet oxygen (Simkin et al 2022; Chen et al 2020). Carotenoids are also involved in a process called “non-photosynthetical quenching” (NPQ), which is conducive to lowering the energy level of singlet excited Chl and to the formation of triple state carotenoids, which can safely release energy via thermal dissipation, preventing the formation of triplet Chl, ROS and singlet oxygen (Simkin et al 2022; Pagels et al 2020a). Three primary xanthophylls present in the antenna complexes of the PSII, namely violaxanthin, antherazanthin, and zeaxanthin, abbreviated as VAZ, are particularly active in NPQ. A reversible interconversion between violaxanthin and zeaxanthin (involving antheraxanthin) occurs, known as the ‘VAZ cycle’, through a series of enzymatic reactions (Simkin et al 2022). Under oversaturating light, violaxanthin is converted to antheraxanthin and subsequently to zeaxanthin, via the effects of the deepoxidase enzyme, with the reverse epoxidation reactions occurring under low light (Mulders et al 2014). The induction of these cycles allows for the avoidance or reduction of cellular damage, as zeaxanthin is used for the dissipation of excess energy from excited chlorophylls during NPQ (Zittelli et al 2023). Thus, the zeaxanthin content of microalgae is usually regulated by light irradiance, and unstressed photosynthetic organisms contain lower zeaxanthin (Eonseon et al 2003). This cycle, which dissipates an excess of light energy as heat occurs in all species of green and brown microalgae (Gupta et al 2021).

**Table 1:** Microalgal strains for carotenoids production

Carotenoid λ_max_ range (nm), color	Microalgal sources
Lycopene λ_max_: 444–505, Red	Not usually observed. Transitory presence
α-carotene λ_max_: 422–473, Pale-Yellow	Green microalgae: *Tetraselmis (suecica*, *chui*), *Dunaniella salina*
β-carotene λ_max_: 425–480, Orange	Green microalgae: *Dunaliella* (*salina*, *bardawil*), *Scenedesmus (almariensis, quadricauda)*, *Tetraselmis suecica*
Astaxanthin λ_max_: 478, Red	Green microalgae: *Haematococcus lacustris*, *Chromochloris zofingiensis* Other microalgae: *Nannochloropsis* (sp., *gaditana)*
Canthaxanthin λ_max_: 466–477, Yellowish-Orange	Green microalgae*: Haematococcus lacustris*, *Chromochloris zofingiensis*, *Chlorella vulgaris*, *Scenedesmus quadricauda*, *Bracteacoccus aggregatus*, *Picochlorum celeri* Other microalgae: *Nannochloropsis gaditana*
Lutein λ_max_: 421–474, Yellowish-red	Green microalgae*: Chlorococcum citriforme*, *Muriellopsis* spp., *Chlorella (Fusca, protothecoides*, *pyrenoidosa*, *vulgaris*, *salina*, *minutissima*, *sorokiniana*), *Scenedesmus* (*quadricauda*, *almeriensis*), *Chromochloris zofingiensis*, *Dunaniella Salina, Tetradesmus obliquus, Botryococcus braunii*
Zeaxanthin λ_max_: 424–480, Yellow	Green microalgae*: Chlorella ellipsoidea*, *Chromochloris zofingiensis*, *Scenedesmus almeriensis* Cyanobacteria*: Synechococcus (sp.*, *elongatus*), *Synechocystis* sp. Red microalgae*: Rhodosorus* sp., *Porphyridium cruentum* Other microalgae: *Nannochloropsis* (*oculata*, *oceánica*), *Phaeodactylum tricornutum*

In contrast to primary carotenoids, secondary carotenoids (e.g. astaxanthin and canthaxanthin) are not bound to the photosystems, as they are only produced when cells are exposed to a stress stimulus, under adverse growth or stress conditions, also known as carotenogenesis conditions (Gupta et al 2021; Mulders et al 2015). They accumulate outside the thylakoids, in cytoplasmic lipid globules, to form a protective layer (Pagels et al 2020a). In some green algae (*Chlorella* and *Scenedesmus)*, secondary carotenoids can also accumulate in the outer cell wall layer (Cano et al 2021). When located in oil droplets outside the photosynthetic apparatus, *β*-carotene accumulates under stress conditions and acts as a secondary pigment (Mulders et al 2014; Shi et al 2020).

**Fig. 2:**
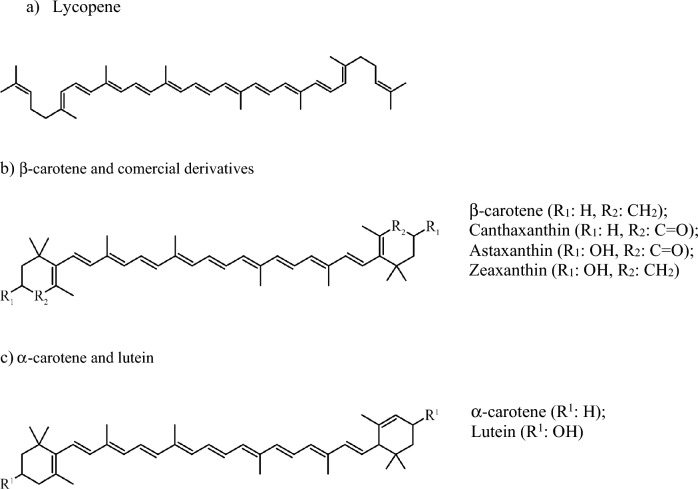
The structure of the carotenoids of main commercial importance

Structurally, most carotenoids have a common 40 carbon structure derived from 8 consecutive isoprene (C_5_H_8_) units, forming a set of conjugated double bonds (Gupta et al 2021) (Fig. 2). Carotenoids are fat-soluble pigments of a yellow, orange or red color (Levasseur et al 2020). Lycopene, which has an acyclic structure, is an intermediate in the biosynthesis of all major carotenes and xanthophyls (Ashokkumar et al 2023). Without altering the molecular formula (C_40_H_56_), microalgae cyclase enzymes mediate the formation of a 9 carbons ring at each end of the lycopene molecule, generating the two main isomers of carotene (α and β). The position of a double bond in one of the rings of these isomers differs, with a final structure incorporating the presence of 10 conjugated double bonds in α-carotene, and 11 in β-carotene (Ashokkumar et al 2023) (Fig. 2). In turn, such carotenes are precursors for the synthesis of microalgal xanthophylls. Lutein and zeaxanthin are formed adding a –OH function via the hydroxylation of α- and β-carotene, respectively (Patel et al 2022; Ashokkumar et al 2023; Kou et al 2020). β-carotene ketolases induce the further formation of the ketocarotenoids, with the introduction of a keto group (C=O) in α-carotene and zeaxanthin, to form canthaxanthin and astaxanthin, respectively (Saini et al 2020) (Fig. 2). Astaxanthin can also be formed by addition of (–OH) via the action of the hydroxylase enzyme on canthaxanthin (Patel et al 2022).

Table 1 shows a non-exhaustive list of the most commonly studied microalgal strains for carotenoid production (Ambati et al 2018; Liang et al 2019; Saini et al 2020; Bourdon et al 2021; Patel et al 2022; Razzak 2024). Among them only two green microalgae, *Dunaliella salina* and *Haematococcus lacustris* (formerly *Haematococcus pluvialis*), are widely used for commercialized microalgal carotenoids, namely β-carotene and astaxanthin, respectively (Rajput et al 2022). As observed in Table 1, the green microalgae possess the largest variety of carotenoid pigments (Mulders et al 2015). Particularly the chlorophyceae family (*Chlorella*, *Dunaliella*, *Haematococcus*, *Chromochloris*) is one particularly important source of carotenoids (Razzak 2024), as it is able to produce both *β*-carotene and important xanthophylls (Levasseur et al 2020).

As a direct or indirect precursor of all carotenoids, lycopene is at least transitorily present in all microalgae, but it is not usually observable in the cells, as it can be immediately converted (Mulders et al 2014). It is however possible to foster lycopene content, by introducing a controlled amount of a cyclase enzyme inhibitor (see section Medium nutrient content and the presence of pigment inducing/inhibiting chemicals).

Regarding carotenes, the β isomer is the preponderant natural form (Gupta et al 2021), found in significant amounts in all microalgae except in the Cryptista and the phycobilin producing type of Dinoflagellata (Mulders et al 2014). Contrastingly, α-carotene is mainly found as the sole isomer in Cryptista, and mixed in with β-carotene in some green microalgae (Patel et al 2022). In such green microalgae (e.g. *Dunaliella* and *Tetraselmis*, Table 1), α-carotene is of minor proportion, and it is postulated that different cyclases are involved in the production specifically of α or β carotene (Gupta et al 2021). Regarding β-carotene production, high concentrations can only be obtained when it accumulates in abundance as a secondary carotenoid in response to stress. Only some species of green microalgae are well known to thusly overproduce such quantities of β-carotene as a secondary pigment (Mulders et al 2014). Despite the commercial success of *D. salina* due to its production of a very high content of β-carotene (up to 98.5% of its total carotenoids, and 13% of its dry biomass (Levasseur et al 2020)), alternatives are currently being studied, with a focus on faster growing microalgae, needing less stringent production conditions, such as *Scenedesmus* or *Tetraselmis* strains (Table 1) (Rajput et al 2022). Regarding astaxanthin, although *Haematococcus lacustris* has proven to be a suitable specie for industrial production, due to its high achievable astaxanthin contents (up to 81% of its total carotenoids, and 7% of its dry weight (Levasseur et al 2020)), alternative producing microalgal species are still studied, as *H. lacustris* can only achieve a low final cell density (e.g., up to 7 gL^−1^) (Liu et al 2014). Not many microalgae can produce astaxanthin, as it contains both oxy- and hydroxyl groups and its production requires the presence of different enzymes (Saini et al 2020). *Chromochloris zofingiensis* (Chlorophyta) has been the most studied alternative for the aim of producing a high content of astaxanthin along with other pigments (Liu et al 2014; Patel et al 2022). Until recently, it was believed that astaxanthin was exclusively found in the Chlorophyta (Mulders et al 2014), however, *Nannochloropsis*, a heterokont microalga in a small class of yellow-green microalgae (Eustigmatophyceae), has been proven to contain a unique combination of photosynthetic pigments, lacking chlorophyll *b* and *c* and including a mixture of xanthophyls, among which are astaxanthin, canthaxanthin, and zeaxanthin (Table 1) (Saini et al 2020; Martins et al 2021). Even if no related production is already implemented, several C*hlorella* have been studied with regard to lutein and zeaxanthin production, together with *Chromochloris zofingiensis* and some *Scenedesmus* species (Saini et al 2020; Levasseur et al 2020). Although lutein is almost exclusively found in green microalgae (Gupta et al 2021; Mulders et al 2014), its isomer zeaxanthin is also found in much more numerous classes including Cyanobacteria and red microalgae, and even in some Heterokontophyta like the diatom *Phaeodactylum tricornutum* (Bourdon et al 2021).

### Phycobiliproteins

Phycobiliproteins (PBPs) are classified into 4 groups, according to their visible absorption spectra, and the relative energy levels of light absorbed. The PBPs which absorb high light energy levels are called phycoerythrins (PE, *λ*_*max*_: 540–570 nm) and phycoerythrocyanins (PEC, *λ*_*max*_: 560–600 nm). In turn, phycocyanins (PC) absorb intermediate energy levels (*λ*_*max*_: 610–620 nm), while allophycocyanins (APC) absorb lower energy levels (*λ*_*max*_: 650–655 nm) (Levasseur et al 2020; Tounsi et al 2023). Commercially, PBPs are usually classified into only two categories, PC and PE, based on their color (blue or red, respectively) (Imchen and Singh 2023). PC industrial microalgal sources mainly include two Cyanobacteria *Arthrospira* sp. and *Aphanizomenon flos-aquae*, while PE sources include the red microalgae *Porphyridium* sp. (Levasseur et al 2020). Recently, the commercial production of PC from *Arthrospira* sp. and PE from *Porphyridium* sp. has increased profoundly (Imchen and Singh 2023).

PBPs have several applications as colorants in the food and cosmetics industries. Actually, the majority (80%) of the PC produced is of medium purity and used in the food industry (Tounsi et al 2023). *Arthrospira* sp. has become the main source of PC, due to the growing maturity of large-scale production, and the consecutive decrease in the price of food-grade PC (Chen et al 2022). Nowadays, algal PC is one of the most extensively used natural blue food colorants, particularly in confectionery, desserts, cake decorations, and icings (Deepika et al 2022; Imchen and Singh 2023). In contrast, commercially available PE is usually of high-purity and is highly costly, which limits its use as a food colorant (Chen et al 2022). Both PC and PE are widely incorporated into cosmetic preparations, for example in anti-ageing, and skin-whitening creams, and eye shadows or eye liners (Deepika et al 2022). Due to their powerful and highly sensitive fluorescent properties, PBPs are also used as markers for certain immunoassays methods, flow cytometry, microscopy and DNA tests. As the most stable PBPs, and one of the world’s brightest fluorophores, PE is the most commonly used fluorescent probe. However, other PBPs are also used for specific applications, like APC, which is a common fluorescent probe used to detect apoptosis (Li et al 2019). Finally, several investigations have reported the biological effects of PBPs, including antioxidant, anti-tumor, anti-inflammatory, and neuroprotective effects, evidencing many potential therapeutic applications for them (Deepika et al 2022; Levasseur et al 2020; Chen et al 2022; Imchen and Singh 2023; Tounsi et al 2023).

PBPs act as auxiliary photosynthetic pigments, by absorbing light not absorbed by chlorophylls, and transferring energy to the photosynthetic reaction center (Deepika et al 2022). Actually, PBPs are the main components of unique large protein complexes called phycobilisomes, which serve as the major light-harvesting antennae in Cyanobacteria, red microalgae, and some chromophytes (Cryptista) (Levasseur et al 2020). Unlike chlorophylls and carotenoids, PBPs are water-soluble and the phycobilisomes are not included inside the thylakoids. Such antennae are attached to the surface of thylakoid membranes in Cyanobacteria and red microalgae, while they are located within the thylakoid lumen in cryptophytes (Tounsi et al 2023). In Cyanobacteria and red microalgae, phycobilisomes are designed to form a specific stacking order of PBPs as the phycobilisome approaches the thylakoid membrane. Actually, light energy is sequentially captured, in an order of decreasing energy, serving as an energetic funnel going from PE, to PC and finally to APC. APC, located in the core of the phycobilisome, finally passes the energy towards the special pair of Chl *a* located in the photosynthetic reaction centers (Tounsi et al 2023; Deepika et al 2022). The phycobilisomes of cryptophytes contain only one type of PBP (PC or PE) and no APC, and Chl *c* acts as an intermediate between this PBP and Chl *a* (Abidizadegan et al 2021). In red algae and Cyanobacteria, APC content is lower than PC and PE content, as APC is only present in the core of the phycobilisome, whereas the latter are positioned all along it (Tounsi et al 2023).

**Fig. 3:**
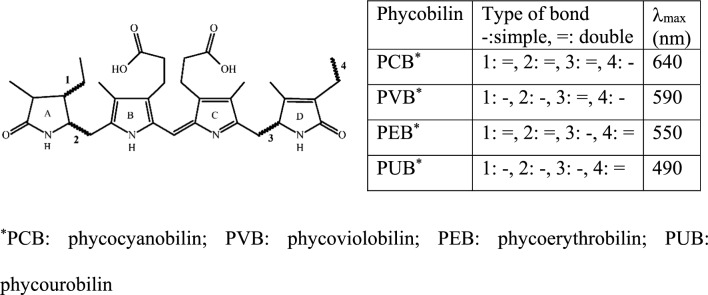
The structure and maximum absorption bands of different phycobilins

Structurally, PBPs are water-soluble pigments which absorb light via special chromophores, the phycobilins. Such phycobilins belong to the tetrapyrrole pigment groups, just like the chlorophylls, but unlike chlorophylls their four pyrroles (usually identified as A, B, C and D) form a non-cyclic chain (Fig. 3) (Saini et al 2020). Four types of phycobilins exist, the phycocyanobilin (PCB), phycoviolobilin (PVB), phycoerythrobilin (PEB), and phycourobilin (PUB), in order of increasing energy levels, and thus decreasing maximum absorption wavelengths order (between 640 and 490 nm) (Li et al 2019; Coulombier et al 2021). PCB and PEB are isomers (C_33_H_38_N_4_O_6_), like PUB and PVB, which have 2 fewer double bonds than the latter (C_33_H_42_N_4_O_6_). Isomers differ in regard to the position of their double bond(s) (Fig. 3). PCB, PVB, PEB, and PUB, absorb red, orange, green and blue-green lights. To form PBPs, large apoproteins are joined to the phycobilins with a thioeter covalent bond generally placed in the A pyrrole ring. PBPs have a molecular weight (Mw) between 220 and 300 kDa (Tounsi et al 2023). PBPs can represent up to 13% of the dry biomass of some microalgae (Levasseur et al 2020)*.*

The most common PBP in Cyanobacteria is PC, whereas in red microalgae it is PE. Although, most red microalgae contain PC, and some Cyanobacteria can synthesize PE or PEC (Pagels et al 2020a). Prefixes have been historically added to PBPs, for reference to their taxonomic origin, with for example a prefix (C-) added when extracted from Cyanobacteria, or (R-) when extracted from red microalgae. Such prefixes are also associated with specific spectral properties (Li et al 2019). APC contains proteins linked to two PCB chromophores and, as a mediator in the transfer of energy collected by phycobilisomes and the photosynthetic reaction centers, it is present in all Cyanobacteria and red microalgae. In comparison to APC, the PEC present in some Cyanobacteria, carries one additional chromophore of CVB. Most commonly found PC in Cyanobacteria (C-phycocyanin) contains three PCB, and most commonly found PC in red microalgae (R-phycocyanin) contains 2 PCB and 1 PEB. In turn, PE present in some Cyanobacteria or red algae have five or six chromophore groups of PEB and/or PUB (Stadnichuk and Tropin 2017).

### Polyphenols

Polyphenolic compounds represent the largest group of secondary metabolites in terrestrial plants, including, among others, water-soluble pigments such as flavonoids and tannins (Levasseur et al 2020; Del Mondo et al 2022; Bhattacharjya et al 2020). Among flavonoids, anthocyanidins and their glycosylated forms (anthocyanins) produce the most diverse palette of colors, responsible for the great variety of floral hues. Anthocyanins present one absorption band within the UV spectrum, and another in the green wavelength, with the latter accountable for the perception of red, purple, or blue colors (Imchen and Singh 2023). Until recently, the presence of anthocyanins in microalgae remained debatable (Del Mondo et al 2022). Although, a red anthocyanin (Cyanidin 3-*O*-(6’’-acetyl-glucoside)) has recently been identified in *Amphidinium carterae* (Dinophyceae), *Navicula* sp. (Bacillariophyceae) and *Tetraselmis suecica* (Chlorophyta) (Zhou et al 2023). Also, the blue pigment marennine, only present in the diatom *Haslea ostrearia,* might have an anthocyanin-based chromophore (Goiris et al 2014). Marennine shows particularly interesting anti-free radical and antioxidant properties (Kolackova et al. 2023).

The absorption spectra of other flavonoids typically consists of two major bands, with one maximum absorption in the UV (band II < 300 nm), and the other in the near-UV electromagnetic spectrum (band I > 300 nm). Some of the non-anthocyanidin flavonoids (isoflavones and flavanones) are colorless for the human eye and cannot be considered as pigments, as they do not absorb light within the visible spectrum (≈380–750 nm) (Taniguchi et al 2023). However, many flavonoids present a bathochromic shift in their absorption spectra. Particularly, the absorption band I of flavones and flavonols has a tail expanding into the visible region (400–450 nm). Such absorption in the blue-purple wavelengths generates their yellow characteristic color (Taniguchi et al 2023). Before the advent of synthetic colorants, flavonoids were widely used to provide yellowish colors in painting lakes and textile dyes (McNab et al 2009). Dye colorants are classified according to the color index constitution number (CI), published by the American Association of Textile Chemists and Colorists. The most common yellow natural dye colorants include flavones (e.g. apigenin (CI 75580); luteolin (CI 75580)), as well as flavonols (e.g. rutin (CI 75730); quercetin (CI 75670); myricetin (CI 75620)) (McNab et al 2009). Beyond their natural coloration ability, flavonoids can also provide UV-protective and antimicrobial properties to cotton, wool, or linen fabrics (Afonso et al 2023). Apart from acting as antimicrobial compounds, flavonoids are highly known for their antioxidant qualities, associated with many therapeutic benefits such as anticancer and anti-inflammatory properties (Cichoński and Chrzanowski 2022; Zhou et al 2023). As such, flavonoids are widely used in pharmaceuticals, cosmetics and food supplements (Bhattacharjya et al 2020). Several studies on the production of polyphenols by macroalgae have been carried out, however few studies of polyphenols production have focused on microalgae, despite recent proof that they contain appreciable levels of them (Levasseur et al 2020; Del Mondo et al 2022). Related genes and polyphenol functions are still not fully known in the case of microalgae (Kolackova et al 2023). Actually, even though phenolics are an important class of pigments, those in microalgae have only been recently identified (Coulombier et al 2021). Microalgae can be an underappreciated origin of such polyphenolic compounds (Zhou et al 2023).

**Fig. 4:**
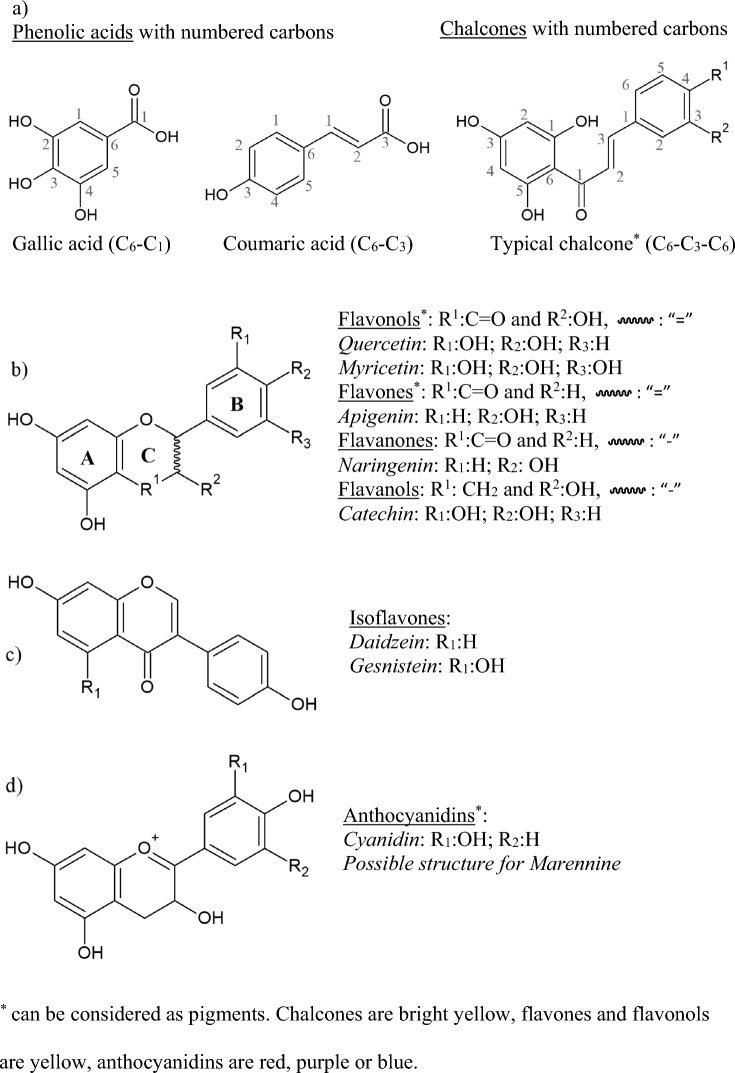
The structure of some typical flavonoids and their precursors. **a** Precursors of flavonoids, and common structure for (**b**) flavonols, flavones, flavanones and flavanols; **c** isoflavones; **d** anthocyanidins

Simple phenolic acids, absorbing light of up to 320 nm, are precursors of polyphenolic pigments. They originate from two different chemical structures, both having a phenol ring (C_6_), but differing in the length of their carbon chain containing the carboxylic group, being derivatives of hydroxybenzoic (C_6_–C_1_, e.g. gallic acid) or hydroxycinnamic acids (C_6_–C_3_, e.g. coumaric acid) (Fig. 4a) (Cichoński and Chrzanowski 2022). In terrestrial plants, gallic acid (also present in microalgae), can be esterified with sugar moieties to form hydrolysable tannins (Zhou et al 2023; Kolackova et al 2023). In turn, the presence of coumaric acid leads to the biosynthesis of flavonoids, including their key precursor chalcones. It is worth noting that coumaric acid itself is derived from phenylalanine or tyrosine aminoacids, as the end-product of the phenylpropanoid pathway, via the action of phenylalanine or tyrosine ammonia-lyases enzymes (PAL, or TAL) (Goiris et al 2014). Many microalgae are able to synthesize coumaric acid, the first precursor of flavonoids, and different types of flavonoids have been found in eukaryotic microalgae and Cyanobacteria (Cichoński and Chrzanowski 2022; Kolackova et al 2023). To date, flavonoids have been detected in two genera of Cyanobacteria (*Nostoc*, *Arthrospira*), as well as in many green microalgae (*Chlamydomonas*, *Chlorella*, *Scenedesmus*, *Dunaliella*, *Tetraselmis*, *Desmodesmus*, *Nannochloris*, *Haematococcus*), and in one red microalga (*Porphyridium purpureum*). Different species of Heterokontophyta such as diatoms (*Phaeodactylum tricornutum*, *Skeletonema marinoi*, *Navicula* sp.), other heterokonts (*Schizochytrium*, *Nannochloropsis*, *Microchloropsis*), as well as one Cryptophyceae (*Proteomonas sulcata*), and a Dinophyceae (*Amphidinium carterae),* also contain flavonoids (Coulombier et al 2021; Zhou et al 2023; Cichoński and Chrzanowski 2022; Kolackova et al 2023; Goiris et al 2014). These discoveries disproved the assumption, enduring until recently, that microalgae lacked the enzymes needed for flavonoids biosynthesis (Goiris et al 2014). Moreover, the total polyphenolic contents of microalgae, usually expressed as gallic acid equivalents (mg_GAE_ g^−1^, (Levasseur et al 2020; Mukherjee et al 2024)), are in the range observed for several vegetables and fruits (Levasseur et al 2020). Specifically, several classes of flavonoids, such as flavones, isoflavones, flavanones, flavanols, and flavonols are found in microalgae (Abidizadegan et al 2021). Content of total flavonoids is usually reported as one of the most common flavonoids, i.e. as quercetin equivalents (mg_QE_ g^−1^, (Cichoński and Chrzanowski 2022)). It can be noted that flavonoids are often found in glycosylated form, with a sugar attached by a glycosidic bond to a carbon or to the oxygen of the flavonoid (C- or O-glycosides, respectively). Rutin is one example of this, as rutin is a rhamnoglucoside of quercetin. In a recent study, total flavonoids in a methanol extract of *Tetradesmus obliquus* (formerly *Scenedesmus obliquus*) (Chlorophyta) were estimated at 5–8 mg_QE_ g^−1^_DW_, and the presence in high amounts of three particular flavonols was noted for the first time, namely myricetin (0.56 mg g^−1^_DW_), rutin (0.75 mg g^−1^_DW_), and quercetin (0.69 mg g^−1^_DW_)*.* Such results matched the quercetin levels found in several fruits and vegetables (Mukherjee et al 2024). A high total flavonoid content was also found in Heterokontophyta (diatoms), and particularly in *Skeletonema* sp. and *Chaetoceros* sp. (5.90, and 4.25 mg_QE_ g^−1^_DW_, respectively), indicating the potential of diatoms for polyphenol pigments production (Bhattacharjya et al 2020). It is worth noting that apigenin is one of the most commonly found flavonoids in microalgal extracts, being detected in many different groups (green microalgae, Cyanobacteria, red microalgae and Heterokontophyta), suggesting that such a flavone may play a well-preserved function in microalgae, which could be linked to UV radiation protection (Del Mondo et al 2022; Goiris et al 2014). Isoflavones (daidzein and genistein) have been found in the diatom *P. tricornutum* and in the Rhodophyta *P. purpureum* (Goiris et al 2014). Regarding flavanols, different catechin derivates or isomers (catechin, epicatechin, galloepicatechin) have been found in red and brown algae, in the diatom *P. tricornutum*, and in some green algae and Cyanobacteria (Del Mondo et al 2022; Zhou et al 2023). Flavanones, and particularly naringenin, seems to be heterogeneously distributed among algal divisions, having only been found in the Haptophyta *Diacronema lutheri* and in the Chlorophyta *Haematococcus lacustris*, and being undetectable in Cyanobacteria, red algae, and diatoms (Del Mondo et al 2022; Goiris et al 2014). It is worth noting that a portion of the polyphenols present in microalgae cannot be directly recovered with water or organic solvents, as it is chemically bounded to microalgal structures and cannot be extracted without a prior process, for example by acid or alkaline hydrolysis. Such bound phenolics content can be higher than that of its free counterparts (Zhou et al 2023). Figure 4 indicates the global structure of flavonoids, including their precursors, phenolic acids and chalcones (Kolackova et al. 2023).

Flavonoids include flavones, flavonols, flavanones, flavanols, isoflavones and anthocyanidins. Structurally, they have a C_6_–C_3_–C_6_ skeleton, with a heterocyclic benzopyran ring (C ring), one fused phenyl ring (A ring, responsible for the II band absorption) and one phenyl (B ring, responsible for the I band absorption) (Fig. 4b) (Taniguchi et al 2023). Flavones, flavonols and flavanones have a keto group in the position R^1^, and differ from each other via a group substitution in R^2^ (OH for flavonols, H for the others), and in the type of one of their carbon–carbon bonds (a simple bond in flavanones, a double in the others). Flavanols are similar to flavonols but with a methyl instead of the keto group in R^1^, and a double carbon–carbon bond replaced by a simple carbon–carbon bond (Fig. 4b). Isoflavones have a different phenyl group position (B ring) to flavones (Fig. 4b, c). Finally, anthocyanidins resemble flavanols, but with a positive charge on their fused ring oxygen (Fig. 4d).

The synthesis of flavonoids starts with the formation of chalcones (containing the basic C_6_–C_3_–C_6_ structure of flavonoids, Fig. 4a), from p-coumaric acid, among other molecules, via the action of the chalcone synthase enzyme. Then, the chalcone isomerase enzyme catalyzes the intramolecular cyclization of chalcones to form flavanones, generating the heterocyclic C ring (Fig. 4b). Flavanones are the precursors of the other flavonoids (Del Mondo et al 2022; Cichoński and Chrzanowski 2022).

The available results suggest that the biosynthetic potential of microalgae is underestimated and that many natural microalgal products remain to be discovered (Kolackova et al 2023). As in other pigments, the concentration of polyphenols and flavonoids within microalgal cells varies in correlation to different factors such as light, nutrient availability, and salinity (Cichoński and Chrzanowski 2022). Ways of increasing pigment contents in microalgae are discussed next.

## Conventional strategies for enhancing pigment content

Several parameters have to be optimized to ensure a maximal pigment content in microalgae. Particularly, an optimum temperature and pH have to be attained for each specific case. However, other critical parameters, such as the quantity and quality of incident light, as well as the availability of nutrients, and the presence of other chemicals in the cultivation medium, like salts, can have a determinant influence on pigment content (Saini et al 2020).

### The effect of light on pigment content

Pigment content and productivity are strongly influenced by the incident light, which can be adjusted in quantitative terms by fixing the light intensity, and the presence and duration of a light/dark cycle (photoperiod) (Pagels et al 2020a, 2020b). Qualitative aspects of the light are also of prime relevance, as the specific spectra of light affects biomass as well as pigments concentrations (Saini et al 2020). However, acclimation strategies and overall response to light are species-specific (Shekh et al 2022).

#### The impact of light intensity and the photoperiod

PC production from *Arthrospira platensis* (Cyanobacteria), cultivated under semi-continuous mode with constant biomass concentrations under different irradiances (635, 980, 1300, 2300 μmol m^−2^ s^−1^), was carried out (Table 2, (Chaiklahan et al 2022)). Interestingly, under continuous illumination, with the lower biomass concentration (optical density (OD):0.4), 2300 μmol m^2^s^−1^ provoked photo-inhibition, with a biomass productivity decrease (from 0.47 (1300 μmol m^2^s^−1^) to 0.29 gL^−1^d^−1^). Such photo-inhibition was absent with higher biomass concentrations, maximal microalgae productivity being attained with 2300 μmol m^2^s^−1^ and OD:0.6 (0.62 gL^−1^d^−1^, Table 2). Conversely, with a higher microalgae concentration (OD:0.8), photo-limitation was observed, as productivity always remained lower than with 0.6 OD (e.g. 0.54 gL^−1^d^−1^, for 0.8 OD, and 2300 μmol m^−2^ s^−1^) (Chaiklahan et al 2022). Thus, the effects of adjustments to light intensity are complex, as the same irradiance can be excessive, suitable, or even limiting for growth, depending on the actual biomass concentration. This is due to the self-shading of light by biomass, which reduces the effective irradiance on cells at higher concentrations. Concurrently, with a given biomass concentration, PC content always increased as irradiance decreased (e.g. for OD:0.8, content gradually rose from 19.0 to 24.4% as irradiance was reduced from 2300 to 635 μmol m^−2^ s^−1^) (Chaiklahan et al 2022). Similarly, with the same applied light intensity, the PC content was always higher with higher biomass concentrations, as more self-shading limited the effective light received by biomass. It has already been reported that above a given minimum needed range, depending on the specific microalgae, decreasing light intensity moderately increases amounts of light-harvesting pigments such as chlorophylls, PBPs and primary carotenoids (mostly lutein) (Saini et al 2020; Zittelli et al 2023; Contreras-Ropero et al 2022; Zheng et al 2022). Acclimation to lower light is achieved via increases in the number of photosystems, this being the means to effectively capture the lower incident light (Zheng et al 2022). Consequently, with a higher number of photosystems, the cellular content of photosynthetic pigments increases (Mulders et al 2014). Noticeably, a drastic PC content reduction was only noted when biomass growth was affected by photo-inhibition (from 16.8 to 4.4% with OD:0.4, between 1300 and 2300 μmol m^−2^ s^−1^) (Chaiklahan et al 2022). Thus, with irradiance stress causing photo-inhibition, PC content is extensively reduced. It has already been observed that subsaturating light can enhance the content of growth-related pigments, such as PBPs, which actively participate in photosynthesis and are located in the photosynthetic apparatus. From a general perspective, an accumulation of photosynthetic pigments occurs under favorable biomass growth conditions, when no stress settings are applied, with no nutrients limitations or the presence of adverse-growth chemicals (Saini et al 2020; Zittelli et al 2023; Mulders et al 2015; Zheng et al 2022). PC content increased when a 12:12 h photoperiod was applied instead of continuous light, increasing from 16.8 to 21.9% for OD:0.4 and 1300 μmol m^−2^ s^−1^, and from 19.9 to 22.0% for OD:0.6 and 2300 μmol m^−2^ s^−1^, respectively (Table 2). The presence of a dark period can facilitate recovery from stress, and is usually sufficiently benefic to enhance PC contents (Shekh et al 2022; Chaiklahan et al 2022; Contreras-Ropero et al 2022). However, the biomass productivity was severely reduced when this photoperiod was applied (Table 2). It is worth noting that more light (without photo-inhibition) and absence of a photoperiod enhanced microalgal biomass productivity, while less light and presence of a photoperiod increased PC contents. Thus, to foster high PC productivity a compromise conducive to elevated biomass productivity should be accepted, allowing for minimal detriment to PC content (Chaiklahan et al 2022; Hsieh-Lo et al 2019). However, biomass growth does not have to take place simultaneously to PBP accumulation. PBP contents can be ultimately fine-tuned. It has been recently reported that a short exposure (90 min) of already grown biomass to a lower light intensity (from 120 to 50 µmol m^−2^ s^−1^) increased PE and PC contents by as much as 65 and 50% (Velasco et al 2023).

**Table 2:** The impact of light conditions on different pigments

Microalgae/mode of cultivation	Pigment(s)/tested conditions	Selected results	Ref
*A. platensis* Zarrouk medium, air feeding, 29 °C, Semi-continuous regime (OD_560nm_: 0.4, 0.6 or 0.8)	PC Irradiance: 635, 980, 1300, 2300 μmolm^−2^ s^−1^ Photoperiod: 24:0 or 12:12	Algal productivities (gL^−1^d^−1^), PC (%) OD 0.4 (1300 μmolm^−2^ s^−1^), 24:0→12:12: 0.47→0.23 gL^−1^d^−1^; 16.8→21.9% OD 0.6 (2300 μmolm^−2^ s^−1^), 24:0→12:12: 0.62→0.33 gL^−1^d^−1^; 19.9→22.0%	Chaiklahan et al. 2022
*P. celeri* Nutrients enriched seawater, 2% CO_2_ feeding, 33 °C, Semi-continuous regime	Chlorophylls Chl *a*, Chl *b* Carotenoids Lutein, Canthaxanthin, VAZ Irradiance: 60→1000 μmolesm^−2^ s^−1^	Biomass (doubling time): 9.5→2.2 h Chlorophyll: 13→5%, Chl *a/b* ratio: 3.2→5.5 Lutein: 2→1%; Canthaxanthin:0.05→1%;VAZ: 0.2→0.6%	Cano et al. 2021
*C. zofingiensis* 6 days cultivation, Kuhl medium, 1.5% CO_2_ feeding, 25 °C, continuous light	Chlorophyll, Astaxanthin Low Light (LL): 80 μmolm^−2^ s^−1^ High Light (HL): 400 μmolm^−2^ s^−1^ Salinity stress (SS): 0.25 M NaCl HL + SS: 400 μmolm^−2^ s^−1^; 0.25 M NaCl	Biomass (gL^−1^d^−1^): LL:1.06;HL:1.65; SS:0.61; HL + SS:1.11 Chlorophyll (% of LL content): HL:50%, HL + SS:10% Astaxanthin: Productivity (mgL^−1^d^−1^): LL:0.8; HL:5.0; SS:1.8; HL + SS:7 Content (mg g^−1^): LL:0.8; HL:3; SS:3; HL + SS:6	Kou et al 2020
*Cyanobium sp.,* air feeding, BG 11 medium with NaCl (10 gL^−1^), Irradiance 200 μmolm^−2^ s^−1^, photoperiod 16:8 h, 21 days	Phycobiliproteins, Carotenoids White light(WL) or red light(RL) Introduction of RL on day 0, 3, 7, 10, 14, or 18, or always under WL	Total phycobiliproteins: 72.0 (WL)→116.7 mgg^−1^ (RL) Total carotenoids: 20.6 (WL)→32.6 mgg^−1^ (RL) Productivity: 21 days WL→10 days WL and 4 days RL Total phycobiliproteins: 9.1→17.0 mgL^−1^d^−1^ Total carotenoids: 2.4→4.5 mgL^−1^d^−1^	Pagels et al. 2020b
*Synechocystis*, BG 11 with NaHCO3 (100 mM)	PC, APC, Chlorophyll WL, targeting lights: PC (620 nm), APC (660 nm), chlorophyll (440 and 680 nm)	Photoinhibition start (wavelength/intensity (μmolm^−2^ s^−1^)) WL/960; 440 nm/348; 620 nm/147; 660 nm/215; 680 nm/400	Bland and Angenent 2016
*A. subsalsa* *Arthrospira* or seawater medium One stage cultivation or two stages cultivation	PC Different light (green, blue, yellow, red, white), Irradiance (0.1–10 k lux), Temperatures (15–40 °C) Two-stages cultivation: 2.5 k Lux under WL at 35 °C(3 d) + 1 k Lux under GL at 25 °C(5 d)	Max biomass content in *Arthrospira* medium: 10 k Lux, 30–35 °C, yellow, red, white light Max phycocyanin content in *Arthrospira* medium: 0.5 K Lux, 25 °C, green light Optimum productivity in *Arthrospira* medium: Two stages: 70 mgL^−1^d^−1^ vs. 40 mgL^−1^d^−1^ at end of 1st stage (WL)	Jiang et al. 2023

Another study shed light on the dynamics of pigments changes related to a strong increase in irradiance level, for the green microalgae *Picochlorum celeri*. Its Chl *a* and *b* content, and that of carotenes such as lutein, canthaxanthin, violaxanthin, antherazanthin, and zeaxanthin were measured regularly from 24 h before, to 48 h after a shift in irradiance from 60 (Low Light (LL)) to 1000 µmol m^−2^ s^−1^ (High Light (HL)). Automated dilutions kept the overall chlorophyll concentration under 0.5 mgL^−1^ to limit biomass self-shading (Cano et al 2021). Biomass increased almost immediately after exposure to HL, the related doubling time going from 9.5 to 2.2 h (Table 2). In LL, *P. celeri* total chlorophyll content amounted to ~ 13% of its biomass, expressed as particulate organic carbon (POC), with a Chl *a*/*b* ratio of 3.2 ± 0.4. After 12 h of HL, its total chlorophyll content was reduced (to 5%), with an upward adjustment to Chl *a/b* ratio (5.5 ± 0.4)*.* As observed in the previous study (Chaiklahan et al 2022), such a total chlorophyll decrease under HL is in accordance with what is expected for photosynthetic growth-related pigments. Stressful conditions (such as HL, and nutrients limitations) usually limit chlorophyll contents (Levasseur et al 2020). Additionally, a higher Chl *a/b* ratio is common in response to higher light intensities (Simkin et al 2022). As the periphery of the light harvesting antenna of green microalgae contains more Chl *b*, a higher Chl *a/b* ratio indicates an antenna size decrease. Under HL, there is no need for the antenna to be larger to support light capture, as a smaller antenna allows for sufficient light penetration and distribution, while limiting photodamage (Cano et al 2021). Lutein, a growth-related pigment, was the dominant carotenoid both under LL and HL, amounting to 65 and 55% of total carotenoids in these respective conditions. Its contents decreased from around 2 to 1% POC with a shift to HL. Contrarily, the content of other carotenoids increased when HL was applied. Particularly, the secondary carotenoid canthaxanthin underwent one of the most rapid content increases after the transition to HL, increasing from 0.05 to 0.1% POC. It has generally been observed that pigments tend to react differently to light intensity in relation to whether they are growth-associated or not. Thus, they require different approaches for their overproduction (Garrido-Cardenas et al 2018; Mulders et al 2015). Non-photosynthetic pigments are generated as a survival strategy under stress conditions which are non-propitious for the growth of microalgae. As such, their content strongly increases under high irradiance, nutrients starvation, and/or presence of an adverse chemical. Under stress conditions, growth related pigments are generally being degraded, while non-growth related pigments are produced in maximum amounts (Mulders et al 2015). This is particularly true in the case of secondary carotenoids (non-thylakoid carotene, astaxanthin, canthaxanthin). Such pigments accumulate in the cells in lipid globules outside the plastids. In accordance with expectations for a secondary carotenoid, more canthaxanthin was produced under HL (stress) conditions. Canthaxanthin was present outside the cell wall, bound to an outer layer or in an extracellular polysaccharide matrix (EPS). It accumulated to as much as 120 mgL^−1^, among which ~ 12% was present in the EPS, where it provided photoprotection against photochemical stress (Cano et al 2021). However, three primary carotenoids, members of the VAZ pool (violaxanthin, antherazanthin, and zeaxanthin), did not adhere to the tendency expected for primary pigments, as their content increased about threefold under HL. Such primary xanthophylls are somehow an exception regarding their behavior under HL. They play a very particular role, and are considered light protecting pigments, as part of the VAZ cycle. In several other studies, the content of pigments involved in the VAZ cycle was also triggered after exposure to HL, or even after nutrient restrictions, with a particular increase of zeaxanthin (Kolackova et al 2023; Zittelli et al 2023). The VAZ cycle pigments, and particularly zeaxanthin, convert excess excitation energy in PSII light collection antenna complexes into heat via NPQ (Coulombier et al 2021; Kolackova et al 2023). Thus, in the case of *Picochlorum celeri*, the operation of the VAZ cycle was a major mechanism used to cope with HL. It is worth noting that zeaxanthin presented the fastest response to the sudden HL exposure, with a strong increase only 2 h after the change to the light conditions (up to sevenfold more % content), followed by a slow decrease. Excess light energy was probably dissipated into heat via NPQ mechanisms preventing damage to the photosystems (Cano et al 2021).

Favorable light conditions can also be combined with other favorable parameters to further enhance pigment production. Recently, the green microalgae *Chromochloris zofingiensis* was cultivated under two different light intensities (LL: 80 and HL: 400 μmol m^−2^ s^−1^), and in presence or in absence of salinity stress (SS) (0.25 M NaCl) (Kou et al 2020). Compared to LL, HL promoted a 1.5-fold higher biomass productivity (1.65 gL^−1^d^−1^), while severely reducing the chlorophyll content (by more than 50%) (Table 2). Again, content of photosynthetic pigments, like chlorophyll, increased to enhance light capture under LL, while they decreased under an excess of light. On the contrary, under HL, astaxanthin content increased 4 times, reaching around 3 mg g^−1^_DW_ (Table 2). The SS treatment also inhibited growth, being conducive to a 40% lower cell density, while obtaining chlorophyll and astaxanthin contents similar to HL. Interestingly, the lower chlorophyll and stronger astaxanthin contents were obtained when the two stress conditions were combined (HL + SS), resulting in a final astaxanthin content of 6 mg g^−1^_DW_, 8.5-fold more than under LL. An analysis of the carotenoids profile clearly indicated that primary carotenoids (such as lutein) declined in response to stress (HL and/or SS conditions). On the contrary, secondary carotenoids, like canthaxanthin, behaved as astaxanthin, accumulating under HL and SS, and even more under combined HL + SS conditions. The total decrease in the amount of primary carotenoids was close to the amount of increased secondary carotenoids, indicating that secondary carotenoids could be derived from the conversion of primary carotenoids. Moreover, the induction of the secondary carotenoids synthesis was concurrent to the synthesis of lipids. Thus HL + SS gave rise to the highest astaxanthin and total fatty acids productivities, which were 7.0 mgL^−1^d^−1^ and 0.51 gL^−1^d^−1^, respectively. It has already been noted that carotenogenesis and lipogenesis generally take place simultaneously under stress conditions (Zittelli et al 2023).

#### The effect of light quality

Pigment productivity is not only influenced by the quantitative aspects of incident light (intensity and presence/absence of photoperiod), but also by qualitative characteristics. However, it is not possible to rely on a universal illumination regime favorable to multiple microalgae (Bland and Angenent 2016). Moreover, a wide discrepancy has been noted between the effects of different lights wavelengths, even among strains of the same species, making it difficult to highlight a specific trend (Contreras-Ropero et al 2022). General considerations presented in the literature, as well as possible reasons for the observed discrepancies are given next with reference to recent studies.

Effects related to light wavelength were analyzed by applying white light (WL) or red light (RL) during the cultivation of a Cyanobacteria (*Cyanobium* sp.) (Table 2, (Pagels et al 2020b)). When applying RL, the biomass growth was faster than under WL, but it stopped on day 11, while continuous growth was observed till day 21 under WL, finally reaching higher biomass contents. Thus, the multichromatic WL allowed for a longer growth period. During the 21 days of growth under WL, the total PBPs and carotenoid productivities reached 9.1 and 2.4 mgL^−1^d^−1^, whereas they reached higher values in 11 days under RL (13.2 and 3.9 mgL^−1^d^−1^, respectively) (Pagels et al 2020b). Actually, total PBP and carotenoids contents were strongly enhanced when applying RL, increasing from 72.0 ± 2.8 to 116.7 ± 5.7 and from 20.6 ± 0.9 to 32.6 ± 1.8 mg g^−1^_DW_, respectively (Table 2). In order to enhance pigments productivity and to increase the growth phase duration, a first WL growth phase, followed by a pigment accumulation phase under RL was optimized. Remarkably, the pigment enhancement was very quick after application of RL, and did not depend on the day that RL started, giving a stable ≈1.6-fold increase of pigments contents. As no additional growth was observed after 14 days, the optimum productivity was observed with 10 days of WL, followed by 4 days of RL, resulting in a productivity of 17.0 ± 0.2 and 4.5 ± 0.2 mgL^−1^d^−1^ for PBPs and carotenoids, respectively. This is significantly higher than the productivities obtained in 21 days under WL (Table 2). Under best productivity conditions, a PBP profile indicated the major presence of PC, followed by APC. It has already been observed that in Cyanobacteria or red microalgae RL can induce a greater accumulation of red absorbing pigments such as PC and APC, while green light (GL) enhances the production of green absorbing pigments such as PE (Stadnichuk and Tropin 2017; Pagels et al 2020a, 2020b). Some Cyanobacteria, containing both PC and PE are even capable to undergo a process known as complementary chromatic adaptation (CCA), drastically changing their biomass color when exposed to GL or RL. When grown under GL, Cyanobacteria like *Oscillatoria sancta,* presents the complementary color (red), while it appears blue-green when grown under RL. Such photoreversible change is due to the accumulation of PC (blue) under RL and PE (red) under GL, allowing cells to maximize light absorption and energy use for photosynthesis (Hsieh-Lo et al 2019). Accordingly, it has been hypothesized that light, within a tolerance limit intensity, targeted for the absorption peak of the photosynthetic pigment can result in augmented pigment content, by delivering the exact wavelength needed by the pigment for its function (Pagels et al 2020b). Remarkably, microalgal contents of carotenoids can also be stimulated by applying a light specifically targeted to carotenoid absorption (blue light (BL)), in both Cyanobacteria (Zittelli et al 2022) and green algae (Zheng et al 2022; Rajput et al 2022). In addition to being easily absorbed by carotenoids, BL is also more energetic than other lights (shorter wavelength), and is believed to foster the formation of ROS, thus enhancing the formation of additional carotenoids to harvest excess energy and prevent damage to the photosynthetic apparatus (Rajput et al 2022). It should also be noted that highly energetic blue and green lights pass easier across the water column than longer wavelengths and are more available at certain depths, which can favor light penetration in big batch cultures (Li et al 2019; Zittelli et al 2023; Patel et al 2022).

However light intensity should be carefully chosen, particularly in the case of monochromatic lights, as was reported when evaluating the growth rates of *Synechocystis* (Cyanobacteria) under different light intensities (0–960 µmol m^−2^ s^−1^) (Bland and Angenent 2016). The effects of WL and a monochromatic light specifically targeting maximum absorptions of PC (620 nm), APC (660 nm) and chlorophyll pigments (440 and 680 nm) were analyzed in regard to biomass growth and pigment content. Although with WL biomass growth rate always increased with light intensity up to the maximum tested intensity (960 µmol m^−2^ s^−1^), a sharp growth rate decrease was noted above a given intensity with different light as a result of photo-inhibition under a monochromatic spectrum. For example, such photoinhibition was estimated to start at 147, 215, and 348 µmol m^−2^ s^−1^ for the 620, 660, and 440 nm lights, respectively (Table 2). It thus appeared that global irradiance is not determinant to ensure that oversaturating or undersaturating light is applied, as the particular spectrum of the light influences the base level from which photoinhibition starts, defining favorable or unfavorable growth conditions. For example, it was observed that with PC targeting light, growth rates were higher than with WL at low light intensities, while they were lower at moderate-high intensities (Bland and Angenent 2016). Many studies apply the same intensity of light when comparing monochromatic and polychromatic lights. However, pigment targeted monochromatic lights, exclusively focused on a narrow band, provide much more energy that is directly available for pigment capture than the same irradiance more evenly distributed across different wavelengths in polychromatic WL. Studies should thus take into account that such focused energy can have a major impact. As a result, the amount of *Synechocystis* biomass achieved after 14 days of cultivation at 250 µmol m^−2^ s^−1^ was equal under 620 nm light and WL (OD_750nm_:4.0), while the content of PC in the cells was lower (however non significantly) (Bland and Angenent 2016). Thus, the use of a specific pigment targeted light is not always beneficial, and it seems that irradiance intensity should be optimized for each different type of light provided. As a result, the selection of a monochromatic light is not a simple task, as each strain has its own specific light tolerance for a given wavelength.

It has been recently noted that the impact of the incident light wavelength on pigment content also strongly depends on other factors such as the culture medium (Jiang et al. 2023). The cultivation of *Arthrospira subsalsa* (formerly *Spirulina subsalsa*) (Cyanobacteria) was carried out under a 1 K lux intensity of different lights (white, yellow (λ_max_: 590 nm), red (λ_max_: 660 nm), green (λ_max_: 525 nm), and blue (λ_max_: 440 nm)), in two growth mediums (one recommended for *Arthrospira* culture, the other composed of seawater), with an initial biomass of 0.1 gL^−1^. In the *Arthrospira* medium, a two-fold higher PC content was obtained under GL (23%) compared to WL (11%), while no significant changes were noted under the other lights. Generally lower PC contents were observed in seawater media, as salt stress could have inhibited photosystem II activity, reducing its PC content. Interestingly, in seawater medium, GL and BL significantly improved the PC content (12 vs. 9.4%). Contrarily to what was observed in the *Arthrospira* medium, a very significant decrease in PC content was noted for RL and yellow light (YL) with seawater medium (6.2 and 4.4%) (Jiang et al 2023). Thus, this indicates that a certain light wavelength can stimulate, maintain, or even reduce pigment content depending on the type of culture media. Regarding biomass, nearly no growth was noted for cultures illuminated with BL and GL, as PC cannot effectively capture such light. Conversely, RL and YL allowed for a better growth in both culture media, surpassing results for WL. It has already been noted in several studies that Cyanobacteria growth is enhanced under RL, as red is highly absorbed by PC, promoting an efficient use of energy and biomass accumulation (Hsieh-Lo et al 2019; Jiang et al 2023). Biomass growth under YL is explained by the great overlap with RL (Jiang et al 2023). Similarly, GL and BL are already known to stimulate Cyanobacteria PC contents, while not promoting growth (Patel et al 2022; Zittelli et al 2023, 2022; Hsieh-Lo et al 2019). Of all lights, BL has the strongest impact on the photophysiology of Cyanobacteria, as BL cannot be absorbed by PC for photosynthesis, and growth is severely dampened. It has been hypothesized that, in order to sustain their activity, cells try to produce more and more PC in an ineffective attempt to reestablish photosystems and incorporate more light energy (Hsieh-Lo et al 2019; Zittelli et al 2022). As neither BL nor GL allow for a substantial biomass growth, they should be applied as a final PC enrichment step, on already grown biomass. The study also tested different temperatures, finding that maximum growth was achieved at a different temperature than maximum PC content (30–35 °C *vs.* 25 °C, respectively) (Jiang et al 2023). For example, in the *Arthrospira* medium, PC content increased from 1.7% of the dry biomass at 15 °C to 7.5% at 25 °C, progressively declining to 4.7% at 40 °C. Regarding irradiance intensity, in the *Arthrospira* medium, the polychromatic light intensity of 0.5 k lux allowed for maximum PC content (11%), while maximum biomass was reached with 10 k lux (1.7 gL^−1^). As in other studies, above a light intensity level that is too low to maintain adequate photosynthesis (0.1 K Lux), lower light intensities allowed for more phycocyanin content, but less biomass growth (Hsieh-Lo et al 2019). In LL, Cyanobacteria accelerate PC synthesis to obtain enough light, while under HL, when the energy is in excess of what microalgae require, a decrease in cellular PC prevents excessive electron formation (Jiang et al 2023). In order to achieve a high PC productivity, both biomass and PC content have to be optimized. A two-stage cultivation, favoring growth over 3 days with high temperature and irradiance (35 °C, 2.5 k Lux, WL) followed by a PC accumulation stage with lower temperature and irradiance under a suitable light (25 °C, 1 K Lux, GL) allowed for a 1.75 higher productivity than at the end of the first phase (70 vs. 40 mgL^−1^d^−1^) (Table 2).

### Medium nutrient content and the presence of pigment inducing/inhibiting chemicals

Several global trends are commonly reported regarding the impact of the mineral medium content, as presented in Table 3. Optimizing media formulation is a traditional approach to increase product yields in microbiology. It is very common to augment non-photosynthetic pigment contents via the incorporation of a stress factor into the mineral medium. The degree of growth inhibition during stress conditions usually determines the pigment production strategy. When inhibition is minor, and enough growth takes place, non-photosynthetic pigment overproduction can be carried-out simultaneously with biomass production, in a continuous process. When too much inhibition is present, and pigment accumulation cannot be realized along with biomass production, a two-step process is prescribed, involving the application of two cultivation conditions, the first promoting algae growth, the second inducing the pigment production (Mulders et al 2014). Several examples involving two-step processes have already been presented in the preceding section in relation to the influence of light on pigment content (Pagels et al 2020b; Jiang et al 2023).

**Table 3:** The impact of mineral medium composition on different pigments

Microalgae/mode of cultivation	Pigment(s)/tested conditions	Selected results	Ref
*H. lacustris* BG-11, 10 days culture, initial 10^5^ cells mL^−1^, 16:8 photoperiod, air supply	Chlorophylls, Carotenes (Astaxanthin) Light: Low Light (LL): 40; High Light (HL): 400 μmolm^−2^ s^−1^ Mineral medium with/without N (+N; -N, respectively)	Final biomass (cells mL^−1^): LL + N: 5.5 10^5^; HL + N: 6.5 10^5^; HL-N and LL-N: no growth Total chlorophylls (pg cell^−1^): LL + N: 78, HL + N and LL-N: 38; HL-N:5 Chl *a/b*: LL + N: 2.15; HL + N: 2.4, LL-N: 3.0, HL-N: 3.7 Astaxanthin (pg cell^−1^): LL + N: 0, HL + N: 250, LL-N: 600; HL-N:1600	Scibilia et al 2015
*D. salina* 10 days cultivation, initial 6000 cells mL^−1^, artificial seawater medium, 25 °C, continuous illumination 150 μmolm^−2^ s^−1^	Lycopene Total Carotenoids Introduction of Nicotine (Ni) on day 5 (0, 20, 50, 100, 200 μM)	Final biomass (10^6^ cells mL^−1^) Ni_0_: 1.5, Ni_20_: 1.7, Ni_50_:0.8, Ni_100_:0.6, Ni_200_: 0.3 Total carotenoids (mgL^−1^): Ni_0_: 3.76, Ni_20_: 3.16, Ni_50_:2.35, Ni_100_:2.17, Ni_200_: 1.58 Lycopene (mgL^−1^): Ni_0_: 0, Ni_20_: 0.68, Ni_50_:0.41, Ni_100_:0.24, Ni_200_: 0	Fazeli et al 2009
*A. maxima* 18 days cultivation, Zarrouk medium (2.5 gL^−1^ NaNO_3_), 0.3% CO_2_, Continuous Fluorescent Light, 28 °C	Total Phenolics/Flavonoids Addition of Phenylalanine (P) (0, 50 and 100 mgL^−1^)	Biomass productivity (mgL^−1^d^−1^): P_0_:37.5, P_50_: 42.6, P_100_:46.7 Total phenolics (mg_GAE_ g^−1^): P_0_:4.51, P_50_: 5.68, P_100_: 7.36 Total flavonoids (mg_QE_ g^−1^): P_0_:1.32, P_50_: 1.54, P_100_: 1.94	El-Baky et al 2009
*T. tetrahele* 11 days cultivation in two-stages (8 + 3d), air injection, Seawater with Conway medium (CM) or Food Factory wastewater (FWW)	Total Phenolics/Flavonoids First stage (8 d) 35 μmolm^−2^ s^−1^ Fluorescent light (F) or 100 μmolm^−2^ s^−1^ LED (L), 25 gL^−1^ salinity Second stage (3 d) increase of salinity (S) (40 gL^−1^), or increase of Light (from 35 to 100 (F) or from 100 to 300 μmolm^−2^ s^−1^ (L))	Biomass: Higher for CM and 35 μmolm^−2^ s^−1^: 2.7 vs. 2.5 gL^−1^ Total phenolics: (6.0–17.6 mg_GAE_ g^−1^) All higher values (11.1 and 17.6 mg_GAE_ g^−1^) with high salt Total flavonoids (mg _QE_ g^−1^) Better results with CM than with FWW: e.g.: with Led light 100 and S 40: 5.12 vs. 0.72 for CM and FWW, respectively	Nezafatian et al 2024
*P. tricornutum,* (Rico et al 2013); *D. tertiolecta *(López et al 2015) 8 days cultivation, Seawater + F/2 medium (control, C), initiating 1 10^7^ (*D. tertiolecta*), and 2 10^7^ cells L^−1^ (*P. tricornutum*)	Total Phenolics/Flavonoids Addition of metals in mineral medium: Fe (+III): 900 nmolL^−1^ Cu (+II): at low (L) and high (H) concentrations: 315 and 790 nmolL^−1^	Final biomass (cells L^−1^) compared to control (*D. tertiolecta*: 1.99 10^8^; *P. tricornutum*: 5.9 10^8^): *P. tricornutum*: Fe: + 471%, Cu (L): −20%, Cu (H): −47.5% *D. tertiolecta:* Fe: + 311%, Cu (L): −15%, Cu (H): −34% Total exuded phenolics per 10^10^ cells compared to control (*D. tertiolecta*: 470 nmol; *P. tricornutum*: 413 nmol): *P. tricornutum*: Fe:-75%, Cu (L): + 12%, Cu (H): + 100% *D. tertiolecta*: Fe: −42%, Cu (L): + 4%, Cu (H): + 40% At Cu (H) increment in: Rutin, Myricetin, Catechin, Epicatechin	López et al 2015 Rico et al 2013

#### The effect of nutrient deprivation

Carbon, nitrogen (N), and phosphorus are among the most essential elements for the growth of autotrophic microalgae, and limiting them can considerably affect the biochemical components of microalgae (Ambati et al 2018; Patel et al 2022). Nitrogen may account for up to 10% of the algal biomass (Liu et al 2014). Reducing or eliminating N from the mineral medium is a usual method of altering microalgae pigments, as a way to produce secondary carotenoids (Shekh et al. 2022; Mulders et al 2015). Particularly, it is very well-established that limitations of N greatly increase the astaxanthin content in some green microalgae, including *Haematococcus lacustris,* which is one of the preferred microorganisms for this purpose, as it can accumulate astaxanthin up to 4%_DW_ (Eonseon et al 2003; Liu et al 2014; Mulders et al 2015; Patel et al 2022). A study took a closer look at astaxanthin accumulation resulting from the cultivation of *H. lacustris* in N-free or normal BG11 media, with LL and HL (40 and 400 µmol m^−2^ s^−1^, respectively) (Table 3, (Scibilia et al 2015)). Although no growth was observed in N-free BG11, the content of astaxanthin reached a much higher level when N starvation complemented HL stress (1600 compared to 250 pg cell^−1^, Table 3). While no astaxanthin is detected in absence of stress, N starvation under LL also allowed for a significant astaxanthin accumulation (Scibilia et al 2015), as N limiting conditions can induce astaxanthin accumulation even without coupling with light stress (Patel et al 2022). It is worth noting that phosphorous depletion can also initiate astaxanthin accumulation (Mulders et al 2015). Additionally, the aforementioned study illustrated that N depletion strongly reduced the total chlorophyll content of *H. lacustris*, while increasing the Chl *a/b* ratio (Table 3, (Scibilia et al 2015)). N is an essential component of proteins, nucleic acids and of some growth-related pigments like chlorophylls and phycobiliproteins. A lack of N prevents growth, and it has already been noted to cause a degradation in chlorophyll, as a way to liberate some N in the medium (Shekh et al 2022; Mulders et al 2015). As previously indicated, an increase of Chl *a/b* is related to an antenna size decrease. Thus, in response to N stress, *H. lacustris* tries to limit the other source of stress (light) by decreasing the antenna size, with, for example, Chl *a/b* increasing from 2.4 to 3.7 with HL. As no growth is possible in the absence of N, industrial production of astaxanthin is usually carried out in two stages, one involving biomass growth, and the other enhancing pigmentation. During the growth phase, without nutrient limitation and under a low irradiance, *H. lacustris* remains in a green, motile growing form. This phase is followed by a red stage, during which *H. lacustris* undergoes a morphological change induced by stress, evolving into resistant cysts which lose mobility and growth capability, but accumulate high amounts of astaxanthin. This second phase involves nutrient deprivation in conjunction with light and/or salinity stresses (Shi et al 2020).

Carotene content augmentation in some green microalgae is realized in response to adverse growth conditions, as for astaxanthin, in a similar manner (Mulders et al 2014; Gong and Bassi 2016). The preferred microalgal source of β-carotene is *Dunaliella salina*, the most halotolerant alga known to date, which can survive in salt concentrations of up to 35% (Shekh et al 2022). *D. salina* can accumulate up to 14%_DW_ of β-carotene in the interthylakoid spaces of the chloroplast stroma, in the form of lipid globules (Cao et al 2023). The accumulation of β-carotene is carried out under various stress conditions such as high salinity, high light intensity and N limitation (Saini et al 2020; Shekh et al 2022). *D. salina* optimal growth salinity is around 22%, while β-carotene content is more abundant with about 33% NaCl (Mobin and Alam 2017). However, growth can be compatible with β-carotene production, and industrial production can be carried out both in two stages (growth followed by pigment accumulation), or in one continuous phase with intermediary stress conditions (Gong and Bassi 2016; Mobin and Alam 2017).

#### The effect of adding an inducing/inhibiting chemical agent

As *D. salina* has a unique ability to produce large amounts of β-carotene, it also generates high amounts of its precursor lycopene. A study evaluated the potential of *D. salina* to produce lycopene, by supplying nicotine, an inhibitor of the enzymatic cyclization of lycopene into carotene, to the mineral medium (Table 3, (Fazeli et al 2009)). As expected, under normal growth conditions, without nicotine, lycopene was not detected, because the lycopene cyclase acts faster than the production of lycopene, transforming all generated lycopene into β-carotene. However, when the mineral medium was supplemented with 20 μM of nicotine, β-carotene content decreased, while lycopene increased (reaching 0.68 mgL^−1^). Higher nicotine concentration (> 50 μM) suppressed microalgal growth, thus progressively damaging cells and reducing both carotene and lycopene production (Table 3). Thus, this study indicates that enzyme inhibitors can be used in the mineral medium to slow down parts of biochemical pathways, forcing the accumulation of desired pigments. However, strong attention should be paid to the dosage of the inhibitors, in order to avoid cell damage, and prevent the blocking of vital biochemical routes. In a more general sense, different types of chemicals can enhance the accumulation of specific pigments, not only by inhibiting biosynthetic pathways, but also by serving as metabolic precursors or by regulating microalgal metabolisms. Details related to the action of such chemical compounds have been reviewed in recent literature (Liang et al 2019).

An example of the effect of adding a chemical precursor, pertaining to polyphenol production, is given in Table 3 (El-Baky et al 2009). Polyphenol content augmentation, unlike that of other chemical groups of microalgal pigments, has been scarcely studied (Levasseur et al 2020; Kolackova et al 2023). However, the next studies presented in Table 3 indicate that an accumulation of polyphenolic compounds can also be stimulated in microalgae, particularly via modifications to the mineral medium composition (Nezafatian et al 2024; El-Baky et al 2009; Rico et al 2013; López et al 2015). As secondary metabolites, polyphenols tend to accumulate under stress conditions, much like secondary carotenoids.

The addition of a metabolic precursor of flavonoids, such as phenylalanine also had a strong impact on their production (El-Baky et al 2009). *Arthrospira maxima* was cultivated in Zarrouk´s medium with the absence or presence of phenylalanine (50 and 100 mgL^−1^). Highest amounts of total phenolic compounds and of flavonoids were reached when the highest concentration of phenylalanine was added to the medium (reaching 7.36 mg_GAE_ g^−1^ and 1.94 mg_QE_ g^−1^ with 100 mgL^−1^ of phenylalanine, vs. 4.51 mg_GAE_ g^−1^ and 1.32 mg_QE_ g^−1^ in the absence of phenylalanine, respectively). Interestingly, in a high-performance liquid chromatography analysis of microalgal extracts, relative chromatographic areas of many phenolic acids (like gallic acid) decreased with higher contents of phenylalanine, while relative areas for flavonoids (like the quercetin pigment) increased, indicating that phenylalanine stimulated flavonoid synthesis (El-Baky et al 2009).

#### The effect of increasing salinity

Increasing the salinity of the medium is a very well-known way to enhance secondary pigment accumulation, which has been largely applied for the production of β-carotene (Mobin and Alam 2017). However, very recently, the effects of salinity and light have been evaluated for the polyphenol production of *Tetraselmis tetrathele* (Chlorophyta), in two different solutions composed of seawater and enriched with Conway medium (CM), or food factory wastewater (FWW) (Nezafatian et al 2024, Table 3). A two-stage cultivation was carried out, involving a first stage of biomass accumulation under a fluorescent light of 35 μmolm^−2^ s^−1^ or a LED light of 100 μmolm^−2^ s^−1^, with a medium salinity of 25 gL^−1^. Under such conditions, the final biomass was slightly higher with the CM illuminated with 35 μmolm^−2^ s^−1^, reaching 2.7 gL^−1^ compared to 2.5 gL^−1^ for other treatments. The second stage involved increasing the salinity to 40 gL^−1^ and maintaining the same lighting conditions, or increasing the light to 100 or 300 μmolm^−2^ s^−1^, in the case of the fluorescent and the LED light, respectively, while maintaining the same salinity. Regarding total phenolic compounds, all the highest values were obtained at high salinity, these being 17.6, 15.0, 14.8, and 11.1 mg_GAE_ g^−1^_DW_ for the FWW medium with 35 μmolm^−2^ s^−1^, the CM medium with 35 and 100, and the FWW medium with 100 μmolm^−2^ s^−1^, respectively. Contrastingly, with lower salinity, total phenolics remained in most cases near 6.0 mg_GAE_ g^−1^_DW_. Thus, salinity stress is a great inducer of phenolic compounds. Regarding flavonoid compounds, the nature of the mineral medium had a strong effect, with much more content resulting from using CM (e.g., for 100 μmolm^−2^ s^−1^ LED and 40 gL^−1^ salinity, 0.72 mg_QE_ g^−1^_DW_ were obtained for FWW vs. 5.12 mg_QE_ g^−1^_DW_ for CM, Table 3).

#### The effect of the presence of metal ions

Two studies dealt with the effects of metals on the flavonoid pigments production of *Phaeodactylum tricornutum* (Rico et al 2013) and *Dunaliella tertiolecta* (López et al 2015). Specifically, ferric and cupric ions were introduced into the medium, at the same concentrations for both studies: 900 nmolL^−1^ for Fe^3+^, and 315 and 790 nmolL^−1^ for Cu^2+^. Fe^3+^ had a completely different effect than Cu^2+^, promoting cell growth, and reducing the total amount of phenolic compounds exuded by cells, both for the diatom and the green microalgae. It has already been reported that iron is an essential micronutrient that can increase the growth rate of microalgae (Rico et al 2013). Contrarily, copper had a negative effect on the growth of both microalgae, even more at the higher tested concentration. Copper had a toxic effect on cells, which was counteracted by a secretion of phenolic compounds. It has been reported that many metals can introduce oxidative stress during microalgae cultivation (Gong and Bassi 2016; Miazek et al 2015). The exuded polyphenolics acted as ligands, as they formed a complex with the metal ions present in the solution, preventing their accumulation within the cells (Rico et al 2013; López et al 2015). The introduction of 790 nmolL^−1^ of Cu^2+^ allowed for a total phenolic secretion of 660 nmol per 10^10^ cells, which is 40% more than the 470 nmol per 10^10^ cells obtained in the absence of copper, for *D. tertiolecta*. An even higher increase was noted for the diatom, moving from 413 to 828 nmol excreted per 10^10^ cells (+100%) with the presence of the same concentration of Cu^2+^ in the medium (Table 3). In regard to the phenolic compounds, more excretion was noted for the flavonols pigments rutin and myricetin, as well as the flavanols catechin and epicatechin. For example, the extracellular production of catechin, the most exuded flavonoid, was increased by 50% in *D. tertiolecta* and by as much as 126% (reaching 284 nmol per 10^10^ cells) in *Phaeodactylum tricornutum*. Thus, polyphenol production in microalgae can be very extensively modified by metal stress.

## Two new trend strategies to enhance pigment contents

In addition to traditional strategies for increasing pigment content in microalgae, several alternatives are currently being explored. Among them, particularly promising results have been obtained with magnetic fields (MFs) or nanoparticles (NPs).

### Magnetic fields

A growing number of studies present the effects of MFs on microalgae (Li et al 2022). Most of them generate the MF with permanent magnets, keeping the magnetic field intensity constant over time (static magnetic fields) (Font et al 2023). Even if the reported MFs intensities vary broadly from some units to as much as 500 milliTesla (mT), the most common studied ranges are between 25–60 mT (Santos et al 2022). Three studies have been selected to illustrate the potential impact of such novel treatment. Table 4 reports the effects of MFs on the cultivation of 3 green microalgae, namely *Auxenochlorella pyrenoidosa* (formerly *Chlorella pyrenoidosa)* and *Tetradesmus obliquus* (Li et al 2022), as well as *Parachlorella kessleri* (formerly *Chlorella kessleri)* (Bauer et al 2017). A Cyanobacteria (*Synechococcus elongatus*) is also reported for the production of PBPs (Nascimento et al 2023).

**Table 4:** New strategies to enhance microalgae pigment contents

Magnetic field enhancement			
Microalgae/mode of cultivation	Pigment(s)/tested conditions	Selected results	Ref
*S. elongatus* BG-11, 30 °C, 15 days culture, initial 0.25 gL^−1^, 12:12 photoperiod, air supply, 50 μmol⋅m^−2^ s^−1^	Chlorophylls, PBPs (APC, PC) With or without Magnetic field (MF): 30 mT	Biomass (gL^−1^): With MF: 0.83; without MF: 0.74 Pigment content (mg g^−1^). Chl *a*: With MF: 11.2; without MF: 10.1; APC: With MF: 2.6; without MF: 0.6; PC: With MF: 2.5; without MF: 1.3	Nascimento et al 2023
*A. pyrenoidosa and T. obliquus* 15 days cultivation, 25 °C, 12/12-h light/dark Light, 100 μmol⋅m^−2^ s^−1^	Chlorophylls (Chl *a* and *b*), Total carotenoids With or without Magnetic field (MF): 30 mT	Biomass compared to control: With MF increase of ≈30% Pigment compared to control (mgL^−1^): Chl *a*: + 26–27%, Chl *b*: no difference Carotenoids: + 31% (*T. obliquus*), no change for A. pyrenoidosa	Li et al 2022
*P. kessleri* 10 days cultivation, 30 °C, 12/12-h light/dark Light, 42 μmolm^−2^ s^−1^,, initial 0.25 gL^−1^	Chlorophylls (Chl *a* and *b*), Total carotenoids With or without Magnetic field (MF): 30 and 60 mT 24 h d^−1^ or 1 h d^−1^	Biomass (gL^−1^): 30 mT: 0.84 (24 h); 1.03 (1 h); 60 mT: 0.96 (24 h); 1.39 (1 h); no MF: 0.76 Pigment content compared to control (mgL^−1^): Chl *a*: (30 mT): + 13% (24 h); + 23% (1 h), 60 mT: −70% (24 h); + 39% (1 h) Chl *b*: (30 mT): + 9% (24 h); + 33% (1 h), 60 mT: −70% (24 h); + 59% (1 h) Carotenoids: (30 mT): + 9% (24 h); + 31.5% (1 h), 60 mT: −73% (24 h); -4% (1 h)	Bauer et al 2017

Nanoparticle enhancement			
Microalgae/mode of cultivation	Pigment(s)/tested conditions	Selected results	Ref
*H. lacustris* 15 days, 12/12-h photoperiod aeration, optimal haematococcus medium, 50 μmolm^−2^ s^−1^, 2 ZnO nanoparticles types chemical (C) (average size 80 nm) and green (G) (30 nm)	Astaxanthin Introduction of ZnO at OD:0.6 (0, 50, 100, and 200 mgL^−1^) for 15 days	Final biomass (ZnO (mgL^−1^), biomass concentration (gL^−1^)): Control: 0, 0.95 Green ZnO: 50, 0.88; 100, 0.75; 200, 0.64 Chemical ZnO: 50, 0.84; 100, 0.66; 200, 0.52 Astaxanthin (ZnO (mgL^−1^), Astaxanthin content (mg g^−1^)) Control: 0, 0.3 Green ZnO: 50, 1.2; 100, 20; 200, .5.8 Chemical ZnO: 50, 0.5; 100, 16.8; 200, 3.8	Nasri et al 2021
*D. salina* 30 days cultivation under 100 μmolm^−2^ s^−1^, (growth) followed by 7 days at 600 μmolm^−2^ s^−1^ (β-carotene production), 22 °C, modified artificial seawater, 12/12-h photoperiod	β-carotene Addition of MoS_2_ (0, 20, 50 100, 200 μg L^−1^)	At 50 μgL^−1^ MoS_2_, compared to the control without NPs: Maximum biomass (gL^−1^): + 33% Carotene concentration (gL^−1^): + 48% Carotene content (% _DW_): + 13.4%	Luo et al 2021

The application of a 30 mT MF did not very significantly affect *S. elongatus* final concentration (0.83 vs. 0.74 mgL^−1^, with and without MF, respectively) (Nascimento et al 2023). Similarly, no significant change could be noted regarding Chl *a* content. Contrastingly, the MF strongly increased PBPs contents, reaching 2.6, and 2.5 mg g^−1^ of APC and PC (87 and 333% higher than the control without MF), respectively (Nascimento et al 2023). Thus, in some cases, MF impacts very specifically one type of pigments.

In another study, the same cultivation conditions were applied to two green microalgae (*Auxenochlorella pyrenoidosa* and *Tetradesmus obliquus*), with and without exposure to a 30 mT MF (Li et al 2022). For both species, at the end of the cultivation period, biomass was significantly enhanced by the MF, achieving a final microalgal concentration of 2.1 vs. 1.6 gL^−1^ for *A. pyrenoidosa* and 1.1 vs. 0.8 gL^−1^·for *T. obliquus* (around 30% more biomass, in both cases, Table 4). For *T. obliquus*, this biomass increase was coupled with a very similar increase in Chl *a* and carotenoids pigments (from 14.72 to 18.77 mgL^−1^ and from 4.36 to 5.70 mgL^−1^), corresponding to 27 and 31% more Chl *a* and carotenoids, respectively. However, for *A. pyrenoidosa*, the biomass increase was only related to a similar increase of Chl *a* (from 17.43 to 21.90 mgL^−1^, i.e. +26%), while final carotenoids concentrations were not significantly increased. As indicated in a recent review, an increase in biomass concentration under a MF could generate a pigment concentration increase, without real composition changes in the cells (Santos et al 2022). Actually, to examine the effect of MFs on cell composition, it seems more appropriate to express pigment contents per g of biomass (Santos et al 2022), instead of the gL^−1^ units often presented (Li et al 2022; Bauer et al 2017). Thus, for the two species of microalgae, the MF induced the same biomass concentration increase in the medium, while only proportionally increasing the carotenoids concentration for *T. obliquus* (Li et al 2022). Thus, as observed in many other studies, the effect of MFs is species dependent, and varies widely according to the type of microalgae to which it is applied (Li et al 2022; Font et al 2023).

In the last study, the cultivation of *Parachlorella kessleri* was carried out under two MF intensities (30 and 60 mT), with MF exposure during the overall operation, or just for 1 h per day (1 h d^−1^) (Bauer et al 2017). For the continuous MF exposure, the final biomass increased with MF intensity, giving final concentrations of 0.96, 0.84 and 0.76 gL^−1^, for 60, 30, and 0 mT (control), respectively (Table 4). Regarding the 1 h d^−1^ exposure, the same behavior was observed, although more biomass was obtained compared to continuous exposure, with 1.39 and 1.03 gL^−1^ for 60 and 30 mT, respectively. Pigments concentrations in the control experiment were 6.4, 2.5 and 1.8 mgL^−1^ for Chl *a*, Chl *b* and total carotenoids. With continuous exposure to 30 mT, some pigment enhancement was noted, with an increase in Chl *a*, Chl *b* and total carotenoids concentrations of 13, 9 and 9%, respectively. When the same intensity was applied for only 1 h per day, Chl *a*, Chl *b* and total carotenoids increased much more (23, 33 and 31.5%, respectively). It has already been noted that the effects of MFs are not necessarily enhanced by a longer exposure, and that, in some cases, short-term exposure can be more efficient than long-term exposure (Chen et al 2023). Actually, exposure time is a key parameter during magnetic treatment, as it can strongly change the effects of MFs (Font et al 2023). However, the continuous MF of 60 mT negatively affected composition, the result being around 70% less concentration for the three pigments. This decrease of pigments was noted on the last 2 days of the 10 days cultivation period (Bauer et al 2017). A pigment concentration decrease has already been noted in other cases of MF application. Actually, the effects of MFs do not occur proportionally to MF intensity, as there is what is known as a specific “window” for each specie of microalgae, where maximum positive enhancement is realized (Bauer et al 2017; Chen et al 2023). It is worth noting that, when the 60 mT MF was applied for only 1 h per day, Chl *a* and Chl b concentrations were again enhanced (by 39 and 59%), while carotenoid accumulation was nearly unchanged, compared to the control (Bauer et al 2017).

To date, no mechanism of action comprehensively explains what has been observed regarding the effects of MFs on microalgae, even if many hypotheses have been formulated (Font et al 2023). MFs produce a certain magnetic and oxidative stress on cells, which can trigger several responses in their metabolism (Santos et al 2022). Such oxidative stress has been found to be proportional to the intensity of the MF (Bauer et al 2017). Effects of the stress induced by MFs may be positive, negative or null, depending on the MF intensity, and exposure time (Santos et al 2022). Additionally, MFs can induce steric modifications in the diamagnetic phospholipids forming the cell membranes, deforming structures and increasing membrane permeability (Chen et al 2023). This augmented permeability could explain a better absorption of nutrients and a better growth under moderate intensities of MFs (Font et al 2023). MFs also interact with charged particles, such as essential ions involved in cell metabolism (Santos et al 2022). Moreover, photosynthesis relies on the movement of charged particles, such as complex electron transfers. MFs are known to influence the energy levels and spin orientations of such electrons (Chen et al 2023).

### Nanoparticles

The potential of NPs for microalgal pigment enhancement is presented in Table 4 in relation to the production of the two most commercial carotenoids, astaxanthin and β-carotene, via the use of their common microalgal sources (Nasri et al 2021; Luo et al 2021). In an astaxanthin producing experiment with *H. lacustris*, two types of Zinc oxide (ZnO) NPs were used, one made from pure chemical products (chemical synthesis), the other incorporating pomegranate peels (green synthesis) (Nasri et al 2021). The main difference between them was the larger particle size of the chemical product in comparison to the green ZNO NPs (an average of 80 vs. 30 nm, respectively). Cultivation was realized with 0 (control), 50, 100 and 200 mgL^−1^ of green or chemical ZnO NPs. Regarding *H. lacustris* biomass, both types of ZnO NPs inhibited biomass growth, the amount of biomass always being lower than for the control. Moreover, with higher concentrations of ZnO, the final biomass was lower, reaching, for the chemical ZnO, values of 0.84, 0.66 and 0.52 gL^−1^ with 50, 100, and 200 mgL^−1^ of NPs, respectively (Table 4). It has already been reported that, with the presence of NPs, algal growth inhibition is related to the shading effect resulting from NP agglomeration around microalgal cell walls (Vargas-Estrada et al 2020). Such an accumulation of NPs on the surface of the cells inhibits microalgal growth by reducing light and nutrient accessibility (Nasri et al 2021; Vargas-Estrada et al 2020; Yuan et al 2023). As presented on Table 4, compared to chemical ZnO, the green ZnO always allowed for higher biomass concentrations (e.g. 0.75 gL^−1^ at 100 mgL^−1^) (Nasri et al 2021). It has already been noted that larger nanoparticles (like chemical ZnO) can lead to stronger shading effects (Vargas-Estrada et al 2020). Regarding astaxanthin content, the same trends occurred with both types of ZnO, much more astaxanthin being present at a concentration of 100 mgL^−1^ (20 and 16.8 mg g^−1^ with the green and chemical ZnO, respectively). Such astaxanthin content (2%) is comparable with that obtained with a standard medium and light adjustments (Nasri et al 2021). Thus, the fine dosage of NPs is extremely important, as strong pigment enhancement is only obtained within a specific concentration range. Moreover, the smaller size NPs (green ZnO) exhibited the best results, with higher biomass concentrations and astaxanthin contents at all applied concentrations (Table 4). *H. lacustris* exposed to ZnO underwent a morphological change, from the green motile stage to the encysted red stage, which can accumulate high amounts of astaxanthin, without the application of stresses such as high light, N limitations, or increased salinity (Nasri et al 2021). A part of ZnO NPs, as is the case with other metallic oxide NPs, can dissolve into toxic ions in the culture medium (Aratboni et al 2023). Moreover, small size NPs can penetrate the cells, entering as ionic metals or getting internalized as a whole (Vargas-Estrada et al 2020). Such metallic NPs and their metallic ions are known to cause stress and increase the levels of ROS in microalgae (Aratboni et al 2023). Particularly, once they are in the cytosol, NPs can agglomerate around cellular organelles, altering processes, and inducing ROS formation (Vargas-Estrada et al 2020). With high doses of metallic NPs present, algal growth can be suppressed (Aratboni et al 2023). Metallic NPs are thus considered as novel stressors, which can affect microalgal metabolism and carotenoid production (Nasri et al 2021; Yuan et al 2023). A non-lethal increase in ROS levels can trigger the synthesis of antioxidant secondary carotenoids such as astaxanthin (Yuan et al 2023). As indicated in the study (Nasri et al 2021), the generation of ROS has been reported to be strongly affected by NPs size and concentration (Yuan et al 2023).

Another study reported the use of NPs of molybdenum disulfide (MoS_2_) for the enhancement of β-carotene production by *D. salina*. For this study, concentrations of NPs were a thousand times much lower than for the aforementioned study (Nasri et al 2021), involving the application of 0, 20, 50, 100 and 200 μg L^−1^ of MoS_2_ (Luo et al 2021). Cultivation for this study involved an initial 30-day growth phase, followed by 7 days of carotene accumulation with higher light. Regarding biomass concentration at the end of the growth phase, the presence of 20 and 200 μgL^−1^ of NPs did not result in a significant change. However, the NPs concentrations of 50 and 100 μgL^−1^ induced more *D. salina* biomass, with the higher values obtained at 50 μgL^−1^, achieving a 33% biomass concentration increase (Luo et al 2021). In contrast to results for the previous study (Nasri et al 2021), biomass can thusly increase in presence of NPs, within an optimum NPs concentration range. Different studies have already indicated that different NPs can significantly improve microalgal growth (Aratboni et al 2023). NPs of MoS_2_, like many metallic NPs, generate a degree of stress, increasing the presence of ROS in microalgal cells (Luo et al 2021). However, microalgae can respond favorably to, and even be stimulated by, a low dose of stress, in a phenomenon known as hormesis, which explains the presence of more biomass with low concentrations of NPs (Vargas-Estrada et al 2020). Thus, MoS_2_ NPs can enhance growth in low concentrations, even if such NPs can have toxic effects in higher concentrations (Luo et al 2021). During the pigment accumulation stage, high light (600 μmol m^−2^ s^−1^) was applied with the presence or absence of 50 μgL^−1^ of MoS_2_ NPs. At the end of the 7-day stage, *β*-carotene content in *D. salina* increased from 0.12 to 0.14 g g^−1^_DW_, an increase of 13.4%, compared to the high light alone. The *β*-carotene concentration in the medium increased even more due to the greater amount of resultant biomass, the final result being a 48% augmentation with the presence of 50 μgL^−1^ of MoS_2_ NPs (Table 4). Thus, the combination of two sources of stress, like high light intensity and the presence of NPs seems to be a promising approach to concurrently overproduce biomass and *β*-carotene (Luo et al 2021).

The effects of NPs on microalgae can vary widely in accordance with the specific structure and composition of NPs. To complement their functionality for generating ROS and stimulating the production of non-photosynthetic pigments, NPs can be designed to fulfill particular tasks. For example, they can improve the incident light utilization by photosystems. Particularly, some NPs can have particularly strong light backscattering properties, thereby redistributing the light within the mineral medium (Vargas-Estrada et al 2020), and even amplifying specific wavelengths (Yuan et al 2023; Aratboni et al 2023). Studies currently evaluate NPs composition, shape, and size in order to improve microalgae light utilization, by regulating intensity and transforming the spectra of poorly absorbed light (Yuan et al 2023). However, when cultivated with the presence of NPs, extracted metabolites can contain trace residues of NPs. The removal of such residues should be taken into account (Yuan et al 2023).

## Conclusions

The present contribution offers a global overview of the nature of microalgal pigments, their properties, and their commercial or potential use. It covers from the fundamentals of all the pigment family groups, including the less explored polyphenols, to some of the most up-to-date concepts. The different available strategies for boosting microalgal pigment contents are discussed based on representative recent research. Global trends have been noted, particularly regarding the different conditions needed for the accumulation of photosynthetic and non-photosynthetic pigments. Content of photosynthetic pigments is enhanced under non-stressful conditions conducive to biomass growth. In contrast, accumulation of secondary pigments (the polyphenols and some carotenoids) takes place under adverse-growth conditions in order to counter the formation of ROS. Application of NPs or MFs can be considered as a new stress condition, which can boost the production of non-photosynthetic pigments, by successfully complementing typical stress conditions (e.g. high light, nutrient depletion). However, special attention should be paid to interactions between cultivation parameters, particularly regarding light irradiance, as the same light intensity could generate growth or stress conditions depending on microalgal concentration, light wavelength, and the type of mineral medium. Thus, to develop commercial microalgal pigment production, elemental studies are still needed, especially to achieve better synergistic interactions between physical, chemical and biological cultivation factors.
